# *Prevotella copri* and microbiota members mediate the beneficial effects of a therapeutic food for malnutrition

**DOI:** 10.1038/s41564-024-01628-7

**Published:** 2024-03-19

**Authors:** Hao-Wei Chang, Evan M. Lee, Yi Wang, Cyrus Zhou, Kali M. Pruss, Suzanne Henrissat, Robert Y. Chen, Clara Kao, Matthew C. Hibberd, Hannah M. Lynn, Daniel M. Webber, Marie Crane, Jiye Cheng, Dmitry A. Rodionov, Aleksandr A. Arzamasov, Juan J. Castillo, Garret Couture, Ye Chen, Nikita P. Balcazo, Carlito B. Lebrilla, Nicolas Terrapon, Bernard Henrissat, Olga Ilkayeva, Michael J. Muehlbauer, Christopher B. Newgard, Ishita Mostafa, Subhasish Das, Mustafa Mahfuz, Andrei L. Osterman, Michael J. Barratt, Tahmeed Ahmed, Jeffrey I. Gordon

**Affiliations:** 1 Edison Family Center for Genome Sciences and Systems Biology, Washington University School of Medicine, St. Louis, MO USA; 2 Center for Gut Microbiome and Nutrition Research, Washington University School of Medicine, St. Louis, MO USA; 3 Department of Pathology and Immunology, Washington University School of Medicine, St. Louis, MO USA; 4 Architecture et Fonction des Macromolécules Biologiques, CNRS, Aix-Marseille University, Marseille, France; 5 Infectious and Inflammatory Disease Center, Sanford Burnham Prebys Medical Discovery Institute, La Jolla, CA USA; 6 Department of Chemistry, University of California, Davis, CA USA; 7 Department of Biotechnology and Biomedicine (DTU Bioengineering), Technical University of Denmark, Lyngby, Denmark; 8 Department of Biological Sciences, King Abdulaziz University, Jeddah, Saudi Arabia; 9 Sarah W. Stedman Nutrition and Metabolism Center, Duke University Medical Center, Durham, NC USA; 10 Duke Molecular Physiology Institute, Duke University Medical Center, Durham, NC USA; 11 Department of Medicine, Duke University Medical Center, Durham, NC USA; 12 Department of Pharmacology and Cancer Biology, Duke University Medical Center, Durham, NC USA; 13 International Centre for Diarrhoeal Disease Research, Bangladesh (icddr,b), Dhaka, Bangladesh

**Keywords:** Microbial communities, Microbiome

## Abstract

Microbiota-directed complementary food (MDCF) formulations have been designed to repair the gut communities of malnourished children. A randomized controlled trial demonstrated that one formulation, MDCF-2, improved weight gain in malnourished Bangladeshi children compared to a more calorically dense standard nutritional intervention. Metagenome-assembled genomes from study participants revealed a correlation between ponderal growth and expression of MDCF-2 glycan utilization pathways by *Prevotella copri* strains. To test this correlation, here we use gnotobiotic mice colonized with defined consortia of age- and ponderal growth-associated gut bacterial strains, with or without *P. copri* isolates closely matching the metagenome-assembled genomes. Combining gut metagenomics and metatranscriptomics with host single-nucleus RNA sequencing and gut metabolomic analyses, we identify a key role of *P. copri* in metabolizing MDCF-2 glycans and uncover its interactions with other microbes including *Bifidobacterium infantis*. *P. copri*-containing consortia mediated weight gain and modulated energy metabolism within intestinal epithelial cells. Our results reveal structure–function relationships between MDCF-2 and members of the gut microbiota of malnourished children with potential implications for future therapies.

## Abstract (web summary)

*Prevotella copri*, together with other microbiota members, plays a key role in mediating the beneficial effects of a gut microbiota-directed complementary food for malnourished children on microbiota and host functions.

## Main

Microbial colonization of the infant gut begins at birth in a process that is influenced by mode of delivery, exposure to maternal microbes from various sources (vaginal, skin, faecal and breast milk), environmental microbes and first foods^1,2^. By the third postnatal year, the process of microbial community assembly (‘maturation’) is largely complete, with communities from healthy children at this age showing configurations that resemble those of adult members of the same family^2–4^. By contrast, undernourished children exhibit delayed development of their microbiota; transplantation of these ‘immature’ communities into germ-free mice produces impairments in growth and metabolism in recipient animals^5,6^. In preclinical models, perturbations in the small intestinal microbiota and protein-deficient diets have both been shown to produce enteropathies that share histologic and pathologic features of environmental enteric dysfunction, a chronic condition affecting undernourished children that is characterized by gut/systemic inflammation and impaired nutrient absorption^7–9^. Taken together, these findings highlight the role of the intestinal microbiota and its collection of genes (microbiome) in fostering healthy postnatal growth.

We previously performed a genome-resolved metagenomic analysis of faecal samples serially collected from 12- to 18-month-old Bangladeshi children with moderate acute malnutrition enrolled in a randomized controlled clinical trial. The trial compared the effects of administering a microbiota-directed complementary food prototype (MDCF-2) versus a ready-to-use supplementary food (RUSF) on host physiology and the composition and expressed functions of the childrens’ microbiomes^10,11^. During the 3 month intervention, MDCF-2 produced significantly greater increases in ponderal growth (defined by rate of change in weight for length (height), expressed as WLZ scores), even though MDCF-2 had a 15% lower caloric density. MDCF-2 also promoted significantly greater increases in levels of plasma proteins positively associated with WLZ, including biomarkers and mediators of musculoskeletal and central nervous system development^10^. We also identified metagenome-assembled genomes (MAGs) whose abundances were associated with WLZ scores. Cellulose, galactan, arabinan, xylan and mannan represent the principal non-starch polysaccharides in MDCF-2 (ref. ^11^). Among the 75 MAGs found to be positively correlated with WLZ, two *Prevotella copri* MAGs dominated the expression of carbohydrate utilization pathways that target MDCF-2 polysaccharides. The expression of these pathways and the faecal concentration of glycan metabolism products were significantly positively correlated with the magnitude of the childrens’ improvement in WLZ^11^. The two WLZ-associated *P. copri* MAGs (Bg0018 and Bg0019) share ten functionally conserved polysaccharide utilization loci (PULs), including seven that are completely conserved. The degree of representation of these seven PULs among the 11 *P. copri* MAGs identified in study participants was predictive of each MAG’s strength of association with WLZ.

*Bifidobacterium longum* subsp. *infantis* (hereafter referred to as *B.* *infantis*) is a prominent early colonizer of the infant gut and a principal consumer of human milk oligosaccharides^12^. Studies of healthy versus malnourished Bangladeshi children showed that *B. infantis* is depleted or absent in the microbiota of infants/children with severe acute malnutrition (SAM). A randomized controlled clinical trial showed that administration of a commercial *B. infantis* strain to infants with SAM improved ponderal growth and reduced levels of biomarkers of gut inflammation^13^. Follow-up preclinical studies revealed that the combination of a Bangladeshi *B. infantis* strain (Bg2D9) and a commercial *B. infantis* strain from a US donor promoted weight gain when introduced into gnotobiotic mice colonized with a pretreatment uncultured faecal microbiota from a Bangladeshi infant who had been a participant in the clinical trial^13^.

In this Article, we use gnotobiotic mice to further examine the role of *P. copri* and other community members, including *B. infantis*, in mediating MDCF-2 metabolism, as well as the effects of MDCF-2 on host physiology, notably weight gain and intestinal function. Defined collections of genome-sequenced bacterial strains, cultured from Bangladeshi children, were sequentially introduced into gnotobiotic female mice (dams), with subsequent transmission of these strains to their pups during the suckling and weaning periods. The strains represented microorganisms whose prominence changes at different stages of postnatal gut microbial community assembly in healthy Bangladeshi children (‘age-discriminatory’ taxa)^4–6,14^. They also included WLZ-correlated bacterial strains selected based on their shared features with MAGs identified in the MDCF-2 clinical study^10,11^. Pups were subjected to the same dietary sequence of exclusive milk feeding (from the dam) followed by weaning onto an MDCF-2 supplemented diet. As well as characterizing changes in microbial community composition and function in response to MDCF-2, we also looked at facets of host physiology. Substantial metabolic investments are needed to support the normal daily replacement of large numbers of gut epithelial cells^15^ as well as the functions they normally express in their differentiating and differentiated states. Therefore, we tested the hypothesis that epithelial cell gene expression and metabolism would be sensitive reporters of functional differences associated with defined consortia with or without *P. copri* MAG-representing strains. We report a central role for *P. copri* strains closely resembling the WLZ-associated MAGs in metabolizing glycans present in MDCF-2, plus their capacity, in the context of other microbiota members such as *B. longum* subsp*. infantis* and MDCF-2, to promote weight gain and influence expression of metabolic functions in enterocytes.

## Results

### Dam–pup transmission of age- and WLZ-associated bacteria

We first designed a defined human gut microbial community that reflected the developing gut microbiota of children enrolled in the clinical study^11^. We selected 20 bacterial strains, 16 of which were cultured from the faecal microbiota of 6- to 24-month-old Bangladeshi children living in Mirpur, the urban slum where the previously reported randomized controlled MDCF-2 clinical trial had been performed (Supplementary Table 1a). They included strains initially identified by the close correspondence of their 16S ribosomal RNA gene sequences to (1) a group of taxa that reflect gut microbiota development in healthy Bangladeshi children^4,14^ and (2) taxa whose abundances had statistically significant associations (positive or negative) with the rate of weight gain (β-WLZ)^6,10^. The relatedness of these strains to the 1,000 MAGs assembled from faecal samples obtained from all participants in the clinical study^11^ was determined by average nucleotide sequence identity (ANI) scores, alignment coverage parameters^16,17^ and their encoded metabolic pathways (Supplementary Table 1b–d). Encoded metabolic pathways for carbohydrate utilization, amino acid and vitamin/cofactor biosynthesis, and fermentation in MAGs and cultured strains were defined by in silico reconstructions; the results are described in the form of ‘binary phenotype’ scores denoting pathway presence or absence^11^.

**Extended Data Fig. 1:**
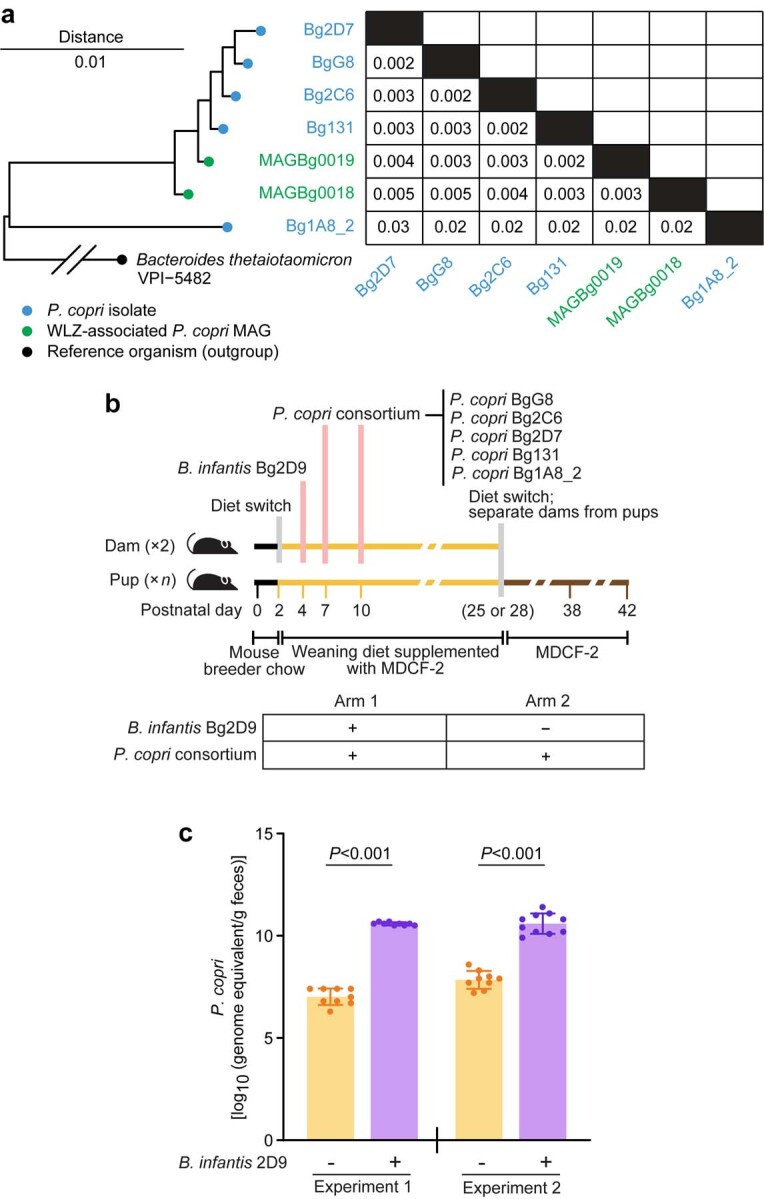
Determining the relationship between *P. copri* colonization efficiency and pre-colonization with *B. longum* subsp. *infantis*. (**a)** Phylogenetic tree of cultured *P. copri* isolates used in the mouse studies described and the two MAGs positively associated with WLZ in the randomized controlled human study. The phylogenetic distance between each pair of comparisons is shown in the matrix. **(b)** Experimental design (*n* = 9 offspring per group in experiment 1 and n = 10 offspring per group in experiment 2). Mice were weaned at P28 and P25 for experiments 1 and 2, respectively. **(c**) Total absolute abundances of *P. copri* strains in fecal samples collected from mice at P42. Mean values ± SD are shown. Each dot represents a separate animal. *P*-values were calculated using a two-sided Mann-Whitney U test and are 4x10^-4^ and 3x10^-4^ for experiments 1 and 2, respectively.

To test how *P. copri* colonization with a strain resembling the two WLZ-associated MAGs, Bg0018 and Bg0019, affected microbial community composition and expressed functions, dietary glycan degradation and host metabolism, we selected the Bangladeshi *P. copri* strain PS131.S11 (abbreviated *P. copri* Bg131). This strain was chosen because of its phylogenetic similarity to Bg0018 and Bg0019 (Extended Data Fig. 1a), the concordance of its metabolic pathway representation with these MAGs (Supplementary Table 1c,d) and its representation of five of the ten functionally conserved PULs shared by Bg0018 and Bg0019. These five PULs are predicted to be involved in degradation of starch, β-glucan, pectin, pectic galactan and xylan (Supplementary Table 1e,f). An additional arabinogalactan-targeting PUL was found adjacent to a conserved PUL-targeting starch, although it did not meet criteria for conservation with the corresponding PUL in MAGs Bg0018 and Bg0019 (Supplementary Table 1f).

To assess the specificity of responses of *P. copri* to MDCF-2, we additionally included an isolate from another *Prevotella* species, *Prevotella stercorea*. No *P. stercorea* MAGs were associated with WLZ in the clinical study, and the cultured isolate did not share any of the PULs present in MAGs Bg0018 and Bg0019 or *P. copri* Bg131. Instead, the PULs in the *P. stercorea* isolate contain glycoside hydrolases that mainly target animal-derived glycans (Supplementary Table 1g). Therefore, we hypothesized this isolate would show lower fitness on the MDCF-2 plant glycan-based diet.

Initial attempts to mono-colonize mice with *P. copri* revealed that it was a poor colonizer on its own (Extended Data Fig. 1b,c). To ensure that *B. infantis* was well represented at the earliest stages of assembly of the defined community so that later colonizers such as *P. copri* could establish themselves, the collection of cultured isolates included two strains of *B. infantis* recovered from Bangladeshi children—*B. infantis* Bg463 and *B. infantis* Bg2D9. The Bg463 strain had been used in our earlier preclinical studies that led to the development of MDCF-2^6,14^. *B. infantis* Bg2D9 had shown greater fitness (absolute abundance) than Bg463 when administered to just-weaned germ-free mice consuming a diet representative of that consumed by 6-month-old children living in Mirpur^13^; this superior fitness was attributed to additional carbohydrate utilization pathways that the strain possesses^13^.

**Fig. 1:**
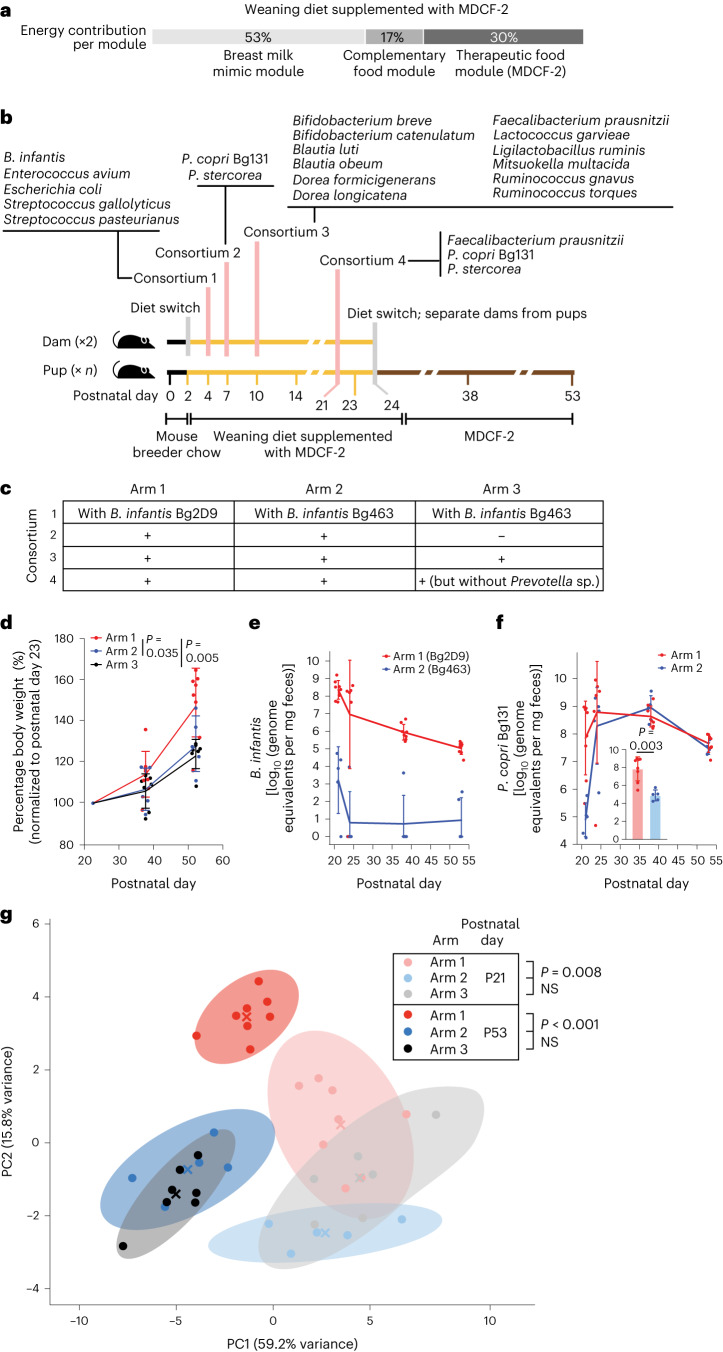
Identifying factors that affect the efficiency of colonization of gnotobiotic dam–pup dyads with *P. copri* in the presence of other cultured age-discriminatory and WLZ-associated bacterial strains and the effects of colonization on pup weight gain. **a**, Energy contribution from different modules of the ‘weaning diet supplemented with MDCF-2’. **b**,**c**, Study design (*n* = 2 dams and 8, 5 and 7 offspring for arms 1, 2 and 3, respectively). **b**, The timing of bacterial colonization of dams and diet switches. **c**, The gavages administered to members of each treatment arm. **d**, Body weights of the offspring of dams, normalized to P23. **e**, Absolute abundance of *B. infantis* Bg2D9 (arm 1) and *B. infantis* Bg463 (arm 2) in faecal samples obtained from pups. **f**, Absolute abundance of *P. copri* in faecal samples collected from pups in the indicated treatment arms at the indicated postnatal time points. Inset: the absolute abundance of *P. copri* in faecal samples collected from pups at P21. **g**, Principal components analysis of absolute abundances of other community members in faecal samples obtained from pups at P21 and P53. Mean values ± s.d. are shown in **d–f**. Each dot in **d–f** represents an individual animal. *P* values were calculated using a linear mixed-effects model (**d**, Methods), a two-sided Mann–Whitney *U* test (**f**, inset) or PERMANOVA (**g**). Centroids are denoted by a coloured ‘X’. Shaded ellipses represent the 95% confidence interval of the sample distribution. Each dot represents an individual animal. Data generated from all of the offspring were used in the analyses shown in **d**–**g**.

We used the 20-strain collection to perform a three-arm, fixed diet study that involved ‘successive’ waves of maternal colonization with four different bacterial consortia (Fig. 1a–c). The sequence of introduction of taxa into dams was designed to emulate temporal features of the normal postnatal development of the human gut community; for example, consortia 1 and 2 consisted of strains that are prominent colonizers of healthy infants/children in the first postnatal year (including the *B. infantis* isolates), while those in consortium 3 are prominent during weaning in the second postnatal year^4–6,14^. This dam-to-pup colonization strategy also helped overcome the technical challenge of reliable delivery of bacterial consortia to newborn pups via oral gavage.

Dually housed germ-free dams were switched from a standard breeder chow to a ‘weaning-diet’ supplemented with MDCF-2 on postpartum day 2, 2 days before initiation of the colonization sequence. This weaning diet was formulated to emulate the diets consumed by children in the clinical trial during MDCF-2 treatment (Methods; Fig. 1a and Supplementary Table 2). Pups in all three arms were subjected to a diet sequence that began with exclusive milk feeding (from the nursing dam) followed by a weaning period where pups had access to the weaning phase diet supplemented with MDCF-2. Pups were weaned at postnatal day 24 (P24), after which time they received MDCF-2 alone ad libitum until P53 when they were euthanized.

To identify factors that promote stable *P. copri* colonization, we compared the effects of the two different *B. infantis* strains, Bg2D9 and Bg463, on *P. copri* and the other cultured strains representing age- and WLZ-associated MAGs. We included *B. infantis* Bg2D9 in consortium 1 of arm 1 of the three-arm experiment and *B. infantis* Bg463 in consortium 1 of arm 2 (Fig. 1b,c). This consortium consisted of five ‘early’ infant gut community colonizers and was administered to dams on postpartum day 4. Dams in these two arms subsequently received the following gavages: (1) on postpartum day 7, *P. copri* and *P. stercorea*; (2) on postpartum day 10, additional age-discriminatory and WLZ-associated taxa and (3) on postpartum day 21, three strains—*P. copri*, *P. stercorea* and *Faecalibacterium prausnitzii* (Fig. 1b). At this last time point, the three strains were given by oral gavage to both the dams and their offspring to help promote successful colonization. To test the effects of *P. copri* colonization, we included arm 3, which was a replicate of arm 2 but without *Prevotella* in consortia 2 and 4. Our rationale for the timing of the first three gavages was based on the diet sequence (gavage 1 of early colonizers at a time (P4) when mice were exclusively consuming the dam’s milk, gavage 2 as the pups were just beginning to consume the human weaning (complementary food) diet, gavage 3 later during this period of ‘complementary feeding’ and the fourth gavage to help to ensure a consistent level of *P. copri* colonization at the end of weaning (and subsequently through the post-weaning period)).

### Effects of *B. infantis* on host weight and microbiota

Gnotobiotic mice colonized with *B. infantis* Bg2D9 exhibited a significantly greater increase in weight gain between P23 (the first time point measured, 2 days after the final gavage) and P53 compared to mice in the two other experimental arms (Fig. 1d and Supplementary Table 3). By contrast, there was no significant difference in weight gain between animals colonized with *B. infantis* Bg463 with or without *Prevotella* species in arms 2 and 3.

To explore whether these differences in weight gain were associated with differences in microbial community composition, we used shotgun sequencing of community DNA to quantify the relative and absolute abundances of all administered strains in faecal samples collected from dams on postpartum days 21, 24 and 35, faecal samples collected from their offspring on P21, P24, P35 and P53, and caecal and ileal contents collected from offspring at P53 (*n* = 2 dams and 5–8 pups analysed per arm; Supplementary Table 4a). Colonization of each consortium member was highly consistent among all animals in each treatment group (Supplementary Table 4b–d). *B. infantis* Bg2D9 successfully colonized pups at P21 in arm 1. By contrast, *B. infantis* Bg463 colonized at 5–8 orders-of-magnitude lower absolute abundance levels in arms 2 and 3 (Supplementary Table 4c). These differences between the groups were sustained through P53. Consistent with the role of *B. infantis* as an early pre-weaning colonizer, both strains decreased in abundance between P21 and P53 (Fig. 1e). Exposure to *B. infantis* Bg2D9 in Arm 1 was associated with levels of *P. copri* colonization that were 3 orders of magnitude greater in the pre-weaning period (P21) than in arm 2 mice exposed to *B. infantis* Bg463 (Fig. 1f and Supplementary Table 4c). Administering the fourth gavage on P21 elevated the absolute abundance of faecal *P. copri* in arm 2 to a level comparable to arm 1; this level was sustained throughout the post-weaning period (P24 to P53) (Fig. 1f and Supplementary Table 4c). This effect of the fourth gavage was also evident in the ileal and caecal microbiota at the time of euthanasia (Supplementary Table 4d).

Based on these results, we directly tested the colonization dependency of *P. copri* on *B. infantis* in two independent experiments whose designs are outlined in Extended Data Fig. 1b. Dually housed germ-free dams were switched from standard breeder chow to the weaning Bangladeshi diet supplemented with MDCF-2 on postpartum day 2. On postpartum day 4, one group of dams was colonized with *B. infantis* Bg2D9, while the other group received a sham gavage. On postpartum days 7 and 10, both groups of gnotobiotic mice were gavaged with a consortium containing five *P. copri* strains. These five *P. copri* strains (Bg131, 1A8, 2C6, 2D7 and G8) were all isolated from faecal samples obtained from Bangladeshi children (Supplementary Table 5). Pups were separated from their dams at the completion of weaning, and their diet was switched to MDCF-2 until euthanasia on P42 (*n* = 9 mice per treatment group; 2 independent control experiments). At this time point, the absolute abundance of *P. copri* in faeces collected from mice that had received *B. infantis* Bg2D9 was 3 orders of magnitude higher than in animals never exposed to *B. infantis* (Extended Data Fig. 1c and Supplementary Table 6).There was no statistically significant difference in weight gain from P23 to P42 between the mono- and bi-colonization groups, although interpreting this finding is at least partially confounded by the increased caecal size and fluid content observed in animals mono-colonized with *P. copri*.

The effects of *B. infantis* on *P. copri* did not generalize to *P. stercorea*. Unlike *P. copri*, the absolute abundance of *P. stercorea* in faeces sampled on P21 and P24 was not significantly different in mice belonging to arms 1 and 2 (Supplementary Table 4c). Before weaning at P24, the absolute abundance of *P. stercorea* was 5 orders of magnitude lower than that of *P. copri*. Throughout the post-weaning period, the absolute abundance of *P. stercorea* remained similar in members of both treatment arms but 2 orders of magnitude below that of *P. copri*.

**Extended Data Fig. 2:**
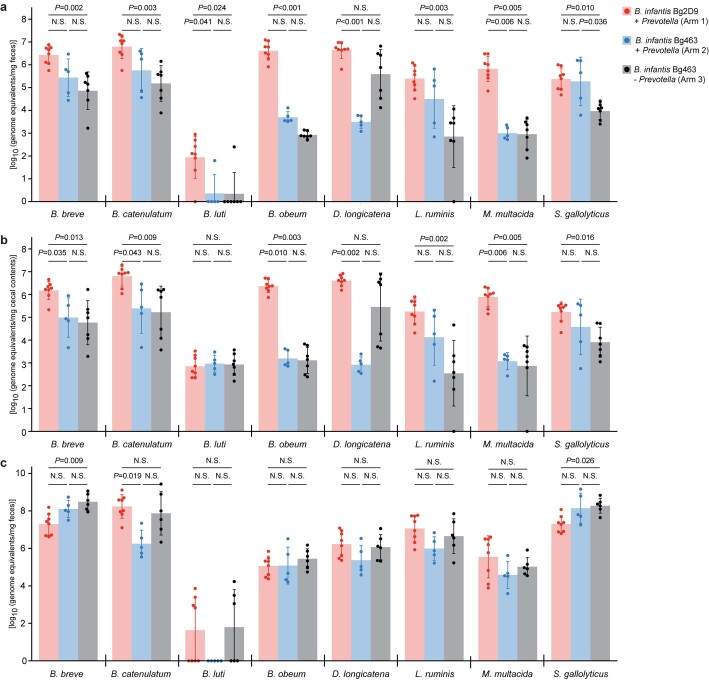
Absolute abundances of other bacterial species in the defined community across different time points and locations. (**a)** Absolute abundances of organisms which were significantly higher with either *B. infantis* Bg2D9 (Arm 1 vs. Arm 2), or with the combination of *B. infantis* Bg2D9 and *Prevotella* (Arm 1 vs. Arm 3) in fecal samples collected at P53 (n = 8, 5, and 7 offspring for arms 1, 2, and 3, respectively). The adjusted *P*-values for *B. obeum* (Arm 1 vs. Arm 3) and *D. longicatena* (Arm 1 vs. Arm 2) are 2x10^-4^ and 7x10^-4^, respectively. (**b, c**) Absolute abundances of the same organisms in cecal contents collected at P53 (panel b) and in fecal samples collected before weaning at P21 (panel c). Mean values ± SD are shown. Each dot represents an individual animal. *P*-values were calculated by the Kruskal-Wallis test followed by post-hoc Dunn’s test with Bonferroni correction. N.S., not significant (*P* > 0.05).

Over the course of the experiment, *B. infantis* Bg2D9 colonization resulted in a faecal community composition that was distinct from that of animals colonized with *B. infantis* Bg463, regardless of their *Prevotella* colonization (Fig. 1g). In animals colonized with *Prevotella* and either of the two *B. infantis* strains (arm 1 versus arm 2), these differences were observed as early as P21 and became more pronounced by the end of the experiment at P53 (Fig. 1g). In animals harbouring *Prevotella*-containing communities, colonization with *B. infantis* Bg2D9 compared to Bg463 significantly increased the fitness of three organisms in the P53 faecal community (*B. luti*, *D. longicatena*, *M. multacida*) and five organisms in the P53 caecal community (*B. breve*, *B. catenulatum*, *B. obeum*, *D. longicatena*, *M. multacida*), while the absolute abundances of the other community members were not significantly affected (Extended Data Fig. 2a–c and Supplementary Table 4c,d).

In animals colonized with *B. infantis* Bg463, the addition of *Prevotella* to the community did not result in significant differences in community composition at either P21 or P53 and only significantly increased the fitness of one organism (*Streptococcus gallolyticus*; Fig. 1g and Extended Data Fig. 2a). However, when comparing arms 1 and 3, the combination of *B. infantis* Bg2D9 and *Prevotella* colonization increased the fitness of a larger set of seven and six organisms in P53 faecal and caecal communities, respectively, including *B. catenulatum*, *Blautia obeum* and *Mitsuokella multacida*—three of the four organisms predicted to be capable of utilizing arabinose (Extended Data Fig. 2a,b); this suggests a potential synergistic interaction between the two organisms in mediating effects on community structure. Based on these results, we concluded that in the context of this preclinical model, (1) *B. infantis* Bg2D9 colonization was an important determinant of microbial community structure, including the fitness of *P. copri*; (2) communities containing *B. infantis* Bg2D9 were associated with augmented weight gain and (3) the temporal profile of community member fitness produced when *B. infantis* Bg2D9 was included more closely resembled that of children in the clinical study who, during the weaning period when MDCF-2 treatment was initiated, all had substantial levels of *P. copri*^11^.

### Microbial metabolism of MDCF-2 glycans

Given these observed differences in microbial community structure, we used ultra-high performance liquid chromatography-triple quadrupole mass spectrometric (UHPLC-QqQ-MS)-based measurements of monosaccharide and linkage content of glycans to analyse the metabolism of MDCF-2 across treatment groups. We sampled the caecum because we wanted to compare microbial gene expression with polysaccharide degradative capacity in a large gut habitat specialized for microbial fermentation^18^.

**Extended Data Fig. 3:**
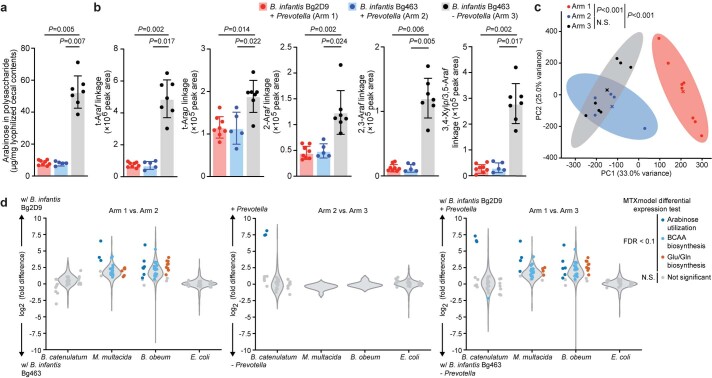
Targeted mass spectrometric and microbial RNA-Seq analyses of consortia of cultured age-discriminatory and WLZ-associated bacteria strains that colonized gnotobiotic mice. Cecal contents collected at the end of the experiment described in Fig. 1a were analyzed. **(a,b)** UHPLC-QqQ-MS-based quantitation of levels of total arabinose (panel a) and arabinose-containing glycosidic linkages (panel b) in cecal glycans collected at P53. Abbreviations: Ara*f*, arabinofuranose; Ara*p*, arabinopyranose; Xyl*p*, xylopyranose. Each dot represents an individual animal (n = 8, 5, and 7 offspring for arms 1, 2, and 3, respectively). Mean values ± s.d. are shown. *P* values were calculated by the Kruskal-Wallis test followed by post-hoc Dunn’s test with Bonferroni correction for panels a and b. (**c**) PCA of profiles of normalized meta-transcriptomic counts (see *Methods*). Centroids are denoted by a colored ‘X’ for each group. *P*-values were calculated by PERMANOVA. (**d**) *MTXmodel* abundance-normalized differential expression analysis of genes involved in specific carbohydrate utilization and amino acid biosynthetic pathways in the four arabinose-utilizing bacteria. Violin plots show the distribution of log_2_ fold-differences for all expressed genes with metabolic pathway annotations in the indicated organism. Dots in panel d represent differential expression test results for individual genes involved in the corresponding pathway and are coloured if their Benjamini-Hochberg adjusted *P-*value is less than 0.1 (see *Methods*). Abbreviations: BCAA, branched-chain amino acid; Glu, glutamate; Gln, glutamine.

While *B. infantis* Bg2D9 colonization was an important determinant of microbial community structure, *Prevotella* colonization drove the degradation of MDCF-2 glycans, regardless of *B. infantis* colonization (Extended Data Fig. 3). *Prevotella* colonization significantly reduced the levels of arabinose in caecal glycans (Extended Data Fig. 3a) and levels of the arabinose-containing linkages t-Ara*p*, t-Ara*f*, 2-Ara*f*, 2,3-Ara*f* and 3,4-Xyl*p*/3,5-Ara*f* (Extended Data Fig. 3b). These differences are supported by the fact that *P. copri* Bg131, unlike *P. stercorea*, contains PULs involved in processing arabinose-containing MDCF-2 glycans: that is, PUL27b specifies carbohydrate active enzymes (CAZymes) known or predicted to digest arabinogalactan, while PUL2 possesses a fucosidase that could target the terminal residues found in arabinogalactan II (Supplementary Table 1f). By contrast, there were no significant differences between animals colonized with *B. infantis* Bg2D9 versus *B. infantis* Bg463 in arms 1 and 2 for any of the monosaccharides or linkages measured (Supplementary Table 7). Together, these results indicate that *Prevotella*-containing communities show more complete degradation of branched arabinans and a greater degree of liberation of arabinose from MDCF-2 glycans.

**Extended Data Fig. 4:**
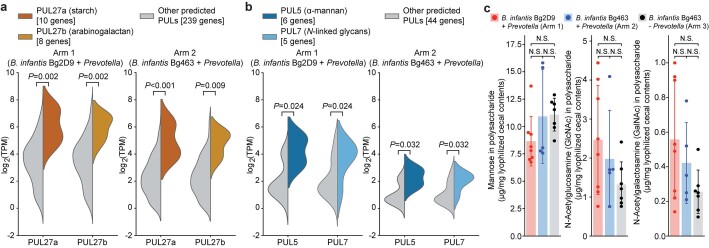
Expression of *P. copri* and *P. stercorea* PULs and targeted mass spectrometric analysis of their predicted targets. (**a,b)** GSEA of expression of PULs shared by *P. copri* Bg131 (panel a) and *P. stercorea* (panel b) in the two *Prevotella-*containing arms of the experiment described in Fig. 1 (n = 8 and 5 offspring for arms 1 and 2 respectively). Benjamini-Hochberg adjusted *P*-values were calculated using GSEA ranking genes by their mean log_2_ TPM across the *Prevotella*-colonized samples in Arms 1 and 2, with each PUL comprising a gene set against the background of all predicted PUL genes. Violin plots show the log_2_ TPM of all genes assigned to any of the PULs in each isolate; plots are split to show the indicated PUL. The exact adjusted *P*-value for PUL27a in Arm 2 (panel a) is 3x10^-4^. (**c**) UHPLC-QqQ-MS-based quantitation of levels of total mannose, N-acetylglucosamine, and N-acetylgalactosamine in cecal glycans collected at P53 (n = 8, 5, and 7 offspring for arms 1, 2, and 3 respectively). Mean values ± SD are shown. Each dot represents an individual animal. *P*-values were calculated by the Kruskal-Wallis test followed by post-hoc Dunn’s test with Bonferroni correction.

To investigate how these changes in glycan utilization are associated with the expressed metabolic functions of community members, we performed microbial RNA sequencing (RNA-seq) on caecal contents (Supplementary Table 8). We first analysed the expression levels of PULs by *P. copri* and *P. stercorea*. In both *Prevotella*-containing arms (arms 1 and 2), *P. copri* PULs with predicted targets of starch and arabinogalactan (PUL 27a and 27b, respectively) were the most significantly enriched for higher levels of expression by gene set enrichment analysis (GSEA) (Methods) (Extended Data Fig. 4a and Supplementary Table 8b).

In contrast to *P. copri*, only two *P. stercorea* PULs with predicted targets of α-mannan and *N*-linked glycans (PULs 5 and 7, respectively) were significantly enriched for higher expression (Extended Data Fig. 4b and Supplementary Table 8b). These two PULs had lower levels of expression than the *P. copri* PULs. Furthermore, unlike the observed reductions in the arabinose content of caecal contents collected from mice colonized with the *Prevotella-*containing consortia, there were no significant differences in mannose, *N*-acetylglucosamine or *N*-acetylgalactosamine, the primary components of glycans targeted by these *P. stercorea* PULs (Extended Data Fig. 4c). These results indicate that *P. copri*, not *P. stercorea*, is responsible for increased liberation of arabinan from MDCF-2 and contributes to degradation of polysaccharides represented in MDCF-2.

We then turned to the rest of the community to determine whether these changes in glycan metabolism had effects on the metabolic responses of other organisms. The *B. infantis* Bg2D9 transcriptome was distinct from *B. infantis* Bg463 (Extended Data Fig. 3c). Consistent with the UHPLC-QqQ-MS-based analysis of caecal glycans, arabinose utilization was among the most upregulated pathways in *B. catenulatum*, *M. multacida* and *B. obeum*—three of the four organisms predicted to be capable of utilizing arabinose (Extended Data Fig. 3d and Supplementary Table 8c,d). These three organisms were also significantly more abundant with *B. infantis* Bg2D9 colonization (Extended Data Fig. 2b). *B. catenulatum* upregulated arabinose utilization genes directly in response to *P. copri* colonization (arm 2 versus arm 3; Extended Data Fig. 3d). By contrast, *M. multacida* and *B. obeum* showed upregulation of arabinose utilization in response to *B. infantis* Bg2D9 colonization (arm 1 versus arm 2; Extended Data Fig. 3d). *M. multacida* and *B. obeum* also showed significant upregulation of almost all their genes involved in biosynthesis of the branched chain amino acids as well as glutamate and glutamine with *B. infantis* Bg2D9 colonization (Extended Data Fig. 3d and Supplementary Table 8d). Together, these findings suggest that while *B. infantis* Bg2D9 colonization affects the abundances of these organisms in the community, glycosidic activities (for example, arabinan degradation) associated with *P. copri* colonization are a primary determinant of their metabolic responses.

### *B. infantis* Bg2D9, *P. copri* and intestinal gene expression

We next tested whether the effects of *B. infantis* Bg2D9 and *P. copri* on microbial community structure and expressed metabolic functions were associated with metabolic changes in epithelial cells in portions of the small intestine that are dedicated to nutrient absorption. For this analysis, we used small intestinal samples from arms 1 and 3 for further analysis. This is because (1) the combination of *B. infantis* Bg2D9 and *P. copri* mediated a set of effects greater than those mediated by either organism alone and (2) successful colonization with both *B. infantis* and *P. copri* before and through the weaning transition at P21 in arm 1 better represented the microbial communities of children in the clinical study^11^. Because of the different *B. infantis* strains used and the greater fitness and greater expression of PULs targeting MDCF-2 glycans shown by *P. copri* but not *P. stercorea*, we refer to arm 1 as ‘with *B. infantis* Bg2D9 and with *P. copri’* and arm 3 as ‘with *B. infantis* Bg463 and without *P. copri*’ for comparisons of the effect of the combination of these organisms.

**Fig. 2:**
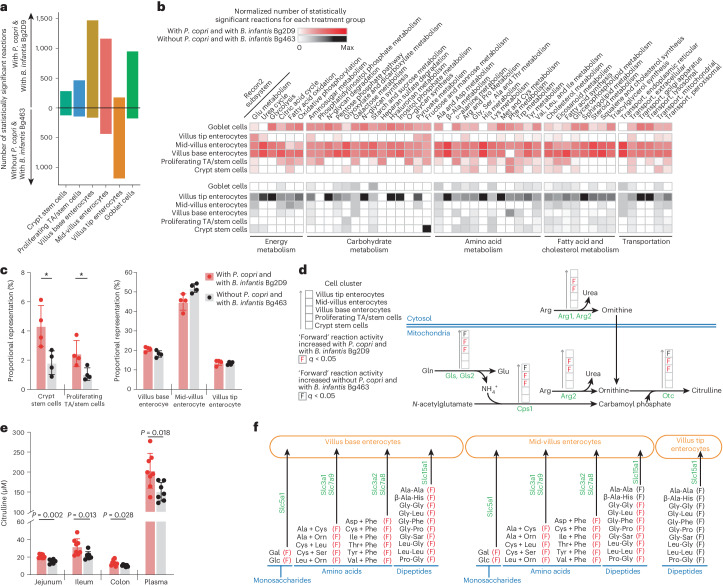
snRNA-seq analysis and targeted mass spectrometric analysis of intestinal tissue and plasma collected from mice containing bacterial communities with or without *P. copri* and two different strains of *B. infantis*. Jejunal tissue samples collected from arm 1 (with *P. copri* and with *B. infantis* Bg2D9) and arm 3 (without *P. copri* and with *B. infantis* Bg463) at the end of the experiment (P53) described in Fig. 1a were analysed (*n* = 4 samples/treatment arm for **a**–**d** and **f**). **a**, The number of Recon2 reactions with statistically significant differences in their predicted flux between mice in Arm 1 and Arm 3. TA, transit amplifying. **b**, The number of Recon2 reactions in each Recon2 subsystem that are predicted to have statistically significant differences in their activities between the two treatment groups. Colours denote values normalized to the sum of all statistically significantly different Recon2 reactions found in all selected cell clusters for a given Recon2 subsystem in each treatment group. **c**, Proportional representation of cell clusters identified by snRNA-seq. Asterisks denote ‘statistically credible differences’ as defined by scCODA (Supplementary Table 10c and Methods). Mean values ± s.d. are shown. **d**, Selected Recon2 reactions in enterocyte clusters distributed along the villus involved in the urea cycle and glutamine metabolism. **e**, Targeted mass spectrometric quantifications of citrulline levels along the length of the gut and in plasma. Mean values ± s.d. and *P* values from the two-sided Mann–Whitney *U* test are shown. Each dot represents an individual animal (*n* = 8 and 7 for arms 1 and 3, respectively). **f**, Effect of colonization with bacterial consortia containing or lacking *P. copri* on extracellular transporters for monosaccharides, amino acids and dipeptides. Ala, alanine; Arg, arginine; Asp, aspartate; Cys, cysteine; Gal, galactose; Glc, glucose; Gln, glutamine; Glu, glutamate; Gly, glycine; His, histidine; Ile, isoleucine; Leu, leucine; Lys, lysine; Met, methionine; Orn, ornithine; Phe, phenylalanine; Pro, proline; Sar, sarcosine; Ser, serine; Thr, threonine; Trp, tryptophan; Tyr, tyrosine; Val, valine. These transporters were selected, and the spatial information of their expressed region along the length of the villus was assigned based on published experimental evidence^29^. Arrows in **d** and **f** indicate the ‘forward’ direction of each Recon2 reaction. The Wilcoxon rank-sum test was used to evaluate the statistical significance of the net reaction scores (**a**, **b**, **d** and **e**) between the two treatment groups. *P* values were calculated from Wilcoxon rank-sum tests and adjusted for multiple comparisons (Benjamini–Hochberg method); *q* < 0.05 was used as the cut-off for statistical significance.

**Extended Data Fig. 5:**
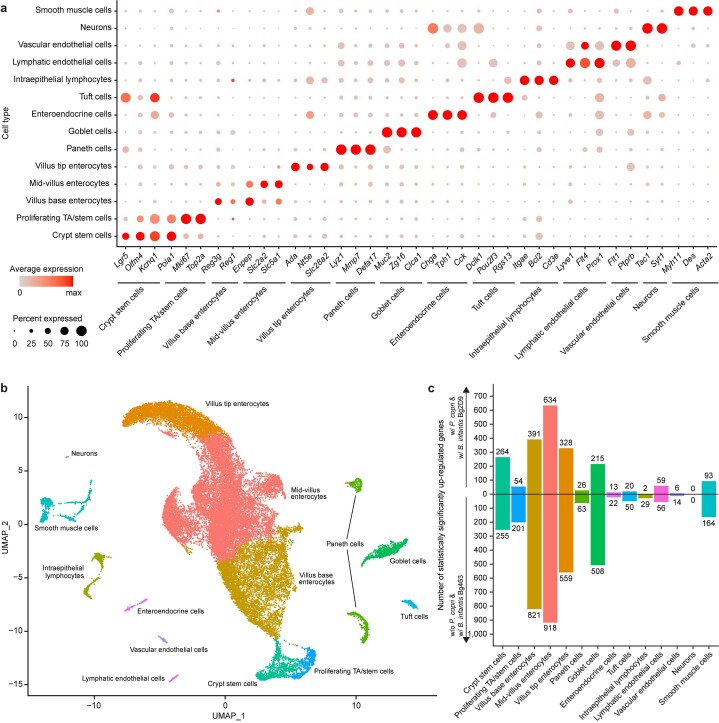
snRNA-Seq analysis of differential intestinal gene expression in mice colonized with bacterial consortia with or without *P. copri*. Jejunal tissue samples collected from ‘w/ *P. copri* & w/ *B. infantis* Bg2D9’ and ‘w/o *P. copri* & w/ *B. infantis* Bg463’ groups at the end of the experiment described in Fig. 1 were analyzed. (**a**) Dot plot of marker gene expression across epithelial cell types. The average expression level and percentage of nuclei that express a given gene within a cell type are indicated by dot color and size, respectively. (**b**) Integrated UMAP plot for all jejunal nuclei isolated from animals in both arms (n = 4 mice/arm). (**c**) The number and directionality of statistically significant differentially expressed genes in each cell cluster.

There were no significant differences in jejunum villus height and crypt depth in mice ‘with *B. infantis* Bg2D9 and with *P. copri’* and ‘with *B. infantis* Bg463 and without *P. copri*’ (*n* = 8 and 7 animals, respectively; Supplementary Table 9). Single-nucleus RNA sequencing (snRNA-seq) was used to investigate whether these two colonization states produced differences in expressed functions in jejunal tissue collected from P53 animals (*n* = 4 per treatment arm; Fig. 2a–c, Extended Data Fig. 5 and Supplementary Table 10). Cell clusters were assigned to the four principal intestinal epithelial cell lineages (enterocytic, goblet, enteroendocrine and Paneth cell) as well as to vascular endothelial cells, lymphatic endothelial cells, smooth muscle cells and enteric neurons (Extended Data Fig. 5a,b). Marker gene analysis allowed us to further subdivide the enterocytic lineage into three clusters: ‘villus base’, ‘mid-villus’ and ‘villus tip’. The majority of all statistically significant differentially expressed genes (3,651 of 5,765; 63.3%) were assigned to these three enterocyte clusters (Extended Data Fig. 5c and Supplementary Table 10b).

**Extended Data Fig. 6:**
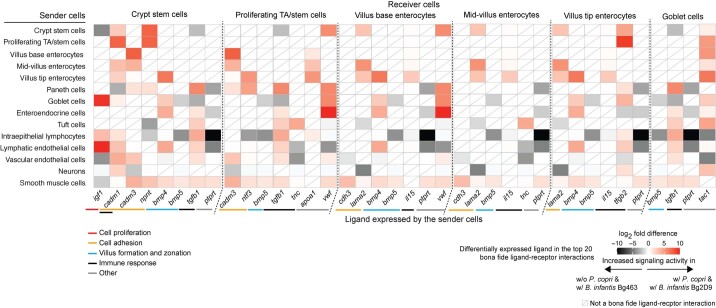
*NicheNet*-based analysis of the effects of *P. copri* colonization on cell-cell signaling activities. Each row represents different sender cell clusters. Each column represents ligands expressed by these sender cells. Cells are colored based on the log_2_-fold difference in expression of ligands in the sender cell clusters between mice in ‘w/ *P. copri* & w/ *B. infantis* Bg2D9’ and ‘w/o *P. copri* & w/ *B. infantis* Bg463’ groups from the experiment described in Fig. 1. Ligands (columns) are grouped based on receiver cell clusters and the indicated functions of downstream signaling pathways in these receiver cells.

We used NicheNet^19^ to identify potential ligand–receptor interactions between receiver and sender cells from our snRNA-seq dataset. We designated six of the epithelial cell clusters (crypt stem cells, proliferating transit amplifying/stem cells, villus base, mid-villus and villus tip enterocytes and goblet cells) as ‘receiver cells’, and all other clusters (both epithelial and mesenchymal) were designated ‘sender cells’. Extended Data Fig. 6 shows ‘bona fide ligand–receptor interactions’^19^ that are altered between the two colonization conditions for each receiver cell cluster. Ligands identified include those known to affect cell proliferation (*igf-1*), cell adhesion (*cadm1*, *cadm3*, *cdh3*, *lama2*, *npnt*), zonation of epithelial cell function/differentiation along the length of the villus (*bmp4*, *bmp5*) and immune responses (*cadm1*, *il15*, *tgfb1*, *tnc*) (Extended Data Fig. 6). Among all receiver cell clusters, crypt stem cells showed the highest number of altered bona fide ligand–receptor interactions. For example, igf-1 is known to enhance intestinal epithelial regeneration^20^. We found that colonization with the *P. copri-*containing consortium was associated with markedly elevated expression of *igf-1* in goblet and lymphatic endothelial sender cells that signal to crypt stem cell receivers.

**Extended Data Fig. 7:**
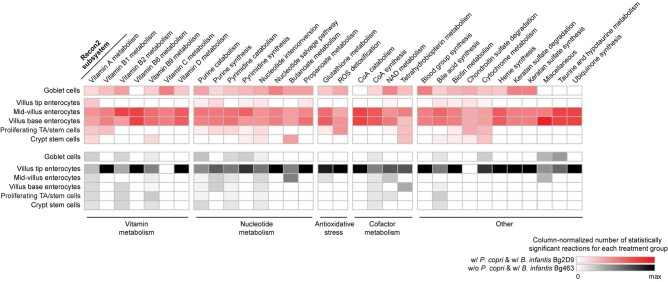
Normalized number of Recon2 reactions in Recon2 subsystems predicted to have statistically significant differences in their activities between mice in the ‘w/ *P. copri* & w/ *B. infantis* Bg2D9’ and ‘w/o *P. copri* & w/ *B. infantis* Bg463’ groups. See legend to Fig. 2b, which shows other affected subsystems, for details.

We subsequently used Compass^21^ and the Recon2^22^ database of metabolic reactions to generate in silico predictions of metabolic flux in different cell clusters: (1) stem cell and proliferating transit amplifying cell clusters positioned in crypts of Lieberkühn, (2) the three villus-associated enterocyte clusters and (3) the goblet cell cluster. Figure 2 shows the predicted metabolic flux differences (Methods) for enterocytes distributed along the length of the villus and in goblet cells. In clusters belonging to the enterocyte lineage, the number of statistically significant differences is greatest in villus base enterocytes and decreases towards the villus tip (Fig. 2a). Compared to mice in arm 3, those in arm 1 had greater predicted increases in the activities of subsystems related to energy metabolism, the metabolism of carbohydrates, amino acids and fatty acids, and various transporters, in villus base and mid-villus enterocytes (Fig. 2b and Extended Data Fig. 7).

While enterocytes prioritize glutamine as their primary energy source, they are also able to utilize fatty acids and glucose. We observed an increase in reactions related to fatty acid oxidation that occur in the villus enterocytes of mice in arm 1 compared to those in arm 3 extended to their crypts of Lieberkühn (Fig. 2b). Fatty acid oxidation has been linked to intestinal stem cell maintenance and regeneration^23^. Mice colonized with *P. copri* and *B. infantis* Bg2D9 exhibited increases in the proportional representation of crypt stem cells and proliferating transit amplifying/stem cells but not in their villus-associated enterocytic clusters (Fig. 2c and Supplementary Table 10c). Compared to mice colonized with *B. infantis* Bg463 and lacking *P. copri*, those colonized with *B. infantis* Bg2D9 and *P. copri* also had predicted increases in energy metabolism in their goblet cells, as judged by the activities of subsystems involved in glutamate metabolism, the urea cycle, fatty acid oxidation and glycolysis (Fig. 2b).

Citrulline is generally poorly represented in human diets; it is predominantly synthesized via metabolism of glutamine in small intestinal enterocytes and transported into the circulation^24^ where it can serve as a quantitative biomarker of metabolically active enterocyte mass. Plasma levels are indicative of the absorptive capacity of the small intestine; they are lower in undernourished children and were increased in Bangladeshi children with moderate acute malnutrition after treatment with MDCF-2^25–27^. Compass predicted that mice harbouring *B. infantis* Bg2D9 and *P. copri* exhibit statistically significant increases in reactions involved in citrulline synthesis in villus base and mid-villus enterocyte clusters (*q* < 0.05 (adjusted *P* value); Wilcoxon ranked sum test; Fig. 2d). Follow-up targeted mass spectrometric analysis confirmed that citrulline was significantly increased in jejunal, ileal and colonic tissue segments, as well as in the plasma of mice in arm 1 compared to arm 3 (Fig. 2e and Supplementary Table 10d).

The presence of *P. copri* and *B. infantis* Bg2D9 was also associated with significantly greater predicted activities in the transport of nine amino acids (including the essential amino acids leucine, isoleucine, valine and phenylalanine), dipeptides and monosaccharides (glucose and galactose) in villus base and mid-villus enterocytes (Fig. 2f). These predictions suggest a greater absorptive capacity for these important growth-promoting nutrients, which are known to be transported within the jejunum at the base and middle regions of villi^24^.

### *P. copri* effects on host metabolism and weight gain

**Extended Data Fig. 8:**
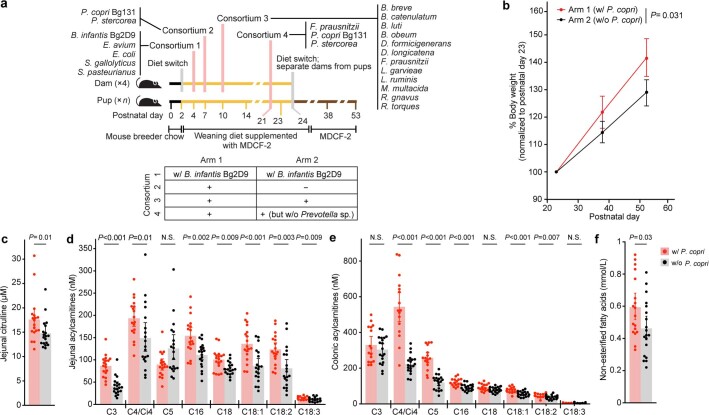
Validating the effects of *P. copri* colonization on postnatal weight gain and host metabolism in gnotobiotic dam-pup dyads. (**a**) Study design (n = 4 dams and 18 and 19 offspring for arms 1 and 2, respectively). (**b**) Body weights of the offspring of dams, normalized to postnatal day 23 [linear mixed effects model (see *Methods*)]. (**c-e**) Targeted mass spectrometric analysis of jejunal citrulline (panel c) and acylcarnitine levels (panel d), plus colonic acylcarnitine levels (panel e). Exact *P*-values for jejunal C3 and jejunal C18:1 (panel d) are 1x10^-5^ and 7x10^-4^, respectively. Exact *P*-values for colonic C4/Ci4, C5, C16, and C18:1 (panel e) are 3x10^-6^, 2x10^-6^, 3x10^-4^, and 7x10^-4^, respectively. (**f**) Plasma levels of non-esterified fatty acids. Each dot represents a single animal. Mean values ± SD are shown for panels b-f. *P*-values were calculated from the linear mixed effect model (panel b) or two-sided Mann-Whitney U test (panels c-f). N.S., *P*-value > 0.05.

We repeated the experiment described above with a larger number of animals (4 dually housed germ-free dams yielding 18–19 viable pups per arm). To examine whether the weight gain phenotype and metabolic alterations observed in the experiment described above could be attributed to the presence or absence of *P. copri* in the microbial community, we administered *B. infantis* Bg2D9 in both arms of this repeat experiment. Outside of this change, the same cultured strains, the same sequence of their introduction and the same sequence of diet switches were applied (Extended Data Fig. 8a). Reproducible colonization of consortium members within each arm was confirmed by quantifying their absolute abundances in caecal samples collected at the time of euthanasia (P53; Supplementary Table 11). As in the previous experiment, animals in the arm containing *P. copri* exhibited significantly greater weight gain between P23 and P53 than those in the no *P. copri* arm (Extended Data Fig. 8b and Supplementary Table 12).

We used targeted mass spectrometry to quantify levels of 20 amino acids, 19 biogenic amines and 66 acylcarnitines in the jejunum, colon, gastrocnemius, quadriceps, heart muscle and liver of the two groups of mice. In addition, we quantified the 66 acylcarnitines in their plasma. The results are described in Supplementary Table 13 and Extended Data Fig. 8c–e. Consistent with the previous experiment, citrulline, a biomarker for metabolically active enterocyte biomass, was significantly elevated in the jejunums of mice belonging to the with-*P. copri* group (Extended Data Fig. 8c and Supplementary Table 13a). We observed significant elevations of acylcarnitines derived from palmitic acid (C16:0), stearic acid (C18:0), oleic acid (C18:1), linoleic acid (C18:2) and linolenic acid (C18:3) in the jejunums of *P. copri*-colonized animals (Extended Data Fig. 8d); these are the major fatty acids found in soybean oil triglycerides^28^, which is the principal source of lipids in MDCF-2. These acylcarnitine chain lengths were found at higher abundances than all other medium or long-chain acylcarnitine species in our jejunal samples, indicating their role as primary dietary lipid energy sources (Supplementary Table 13b). Elevation of these species suggests increased transport and β-oxidation of long-chain dietary lipids in the jejunums of the *P. copri-*colonized animals.

Analysis of colonic tissue showed significant elevation of C16:0, C18:1 and C18:2 acylcarnitines in *P. copri*-colonized animals, suggesting that β-oxidation is also elevated in tissue compartments not directly involved in lipid absorption (*P* < 0.01; Mann*–*Whitney *U* test) (Extended Data Fig. 8e and Supplementary Table 13b). This finding was matched by a significant elevation in plasma levels of non-esterified fatty acids in *P. copri*-colonized animals, which would support fatty acid β-oxidation in peripheral tissues (Extended Data Fig. 8f and Supplementary Table 13c). In addition, colonic (and jejunal) levels of C3 and C4 acylcarnitines known to be derived from branched-chain amino acid catabolism were significantly elevated in the *P. copri*-colonized animals (Extended Data Fig. 8d,e and Supplementary Table 13b).

### *P. copri* colonization and weight gain with another diet

**Extended Data Fig. 9:**
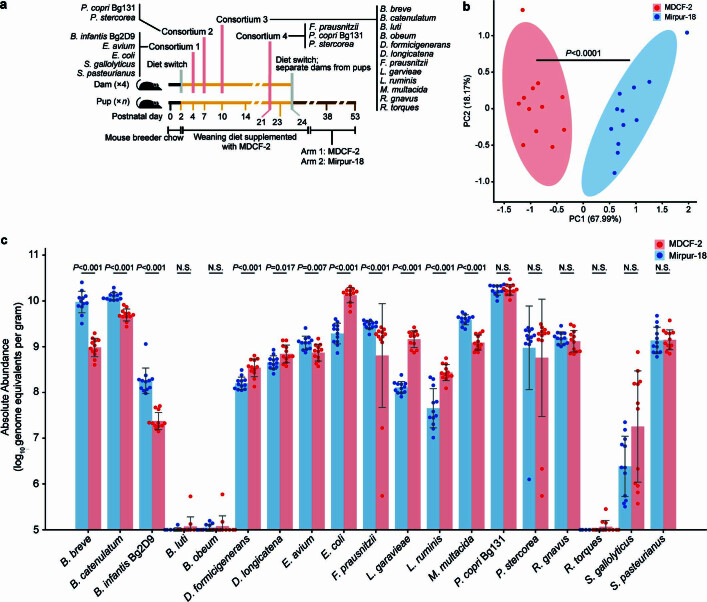
Evaluating the effect of diet on the defined community in gnotobiotic dam-pup dyads. **(a)** Experimental design (n = 2 dams and 12 offspring/diet treatment). **(b)** Principal component analysis showing the significant differences in community structure in the cecums of mice euthanized on P53 (*P* = 1x10^-5^; PERMANOVA). Ellipses represent 95% confidence intervals. **(c)** Absolute abundances of the defined community members in cecal contents at P53. Exact *P*-values for *B. breve*, *B. catenulatum*, *B. infantis* Bg2D9, *D. formicigenerans*, *E. coli*, *F. prausnitzii*, *L. garavieae*, *L. ruminis*, and *M. multacida* are 4×10^-5^, 4×10^-5^, 4×10^-5^, 6×10^-4^, 4×10^-5^, 2×10^-4^, 4×10^-5^, 1×10^-4^, and 4×10^-5^, respectively. *P*-values were calculated by PERMANOVA (panel b) and a two-sided Mann-Whitney U test (panel c). N.S., *P*-value > 0.05. Mean values ± SD are shown. Each dot represents an individual animal.

We next looked at the effects of *P. copri* colonization in the context of a ‘control’ diet representative of that typically consumed by 18-month-old Bangladeshi children living in Mirpur (‘Mirpur-18 diet’)^4^. The design was similar to that used for the experiments described in Fig. 1 and Extended Data Fig. 8 with two exceptions: (1) *B. infantis* Bg2D9 was used in both groups and (2) on P24, pups from different litters were mixed and randomly assigned to two diet treatment groups, MDCF-2 and Mirpur-18 (*n* = 2 dams and 12 pups per group). The absolute abundances of community members were quantified in caecal contents collected at the time of euthanasia on P53. While the absolute abundance of *P. copri* Bg131 was not significantly different between the two diet groups, there were statistically significant differences between the abundances of 11 of the 19 community members (Extended Data Fig. 9). Nonetheless, we proceeded to test whether the increased weight gain phenotype associated with the presence of *P. copri* in the community was evident in the Mirpur-18 diet context. To do so, we repeated the dam-to-pup microbial transmission experiment where all animals were weaned onto the Mirpur-18 diet but where one group had received *P. copri* Bg131 (*n* = 8 animals) and the other had not (*n* = 9). All animals were euthanized on P53. *P. copri* successfully colonized mice and was maintained throughout the experiment at levels comparable to previous experiments (10.4 ± 0.1 log_10_ genome equivalents per gram of caecal contents at P53). Importantly, there was no statistically significant difference in weight gain between the two groups (*P* = 0.297; linear mixed-effects model (Methods)). These findings provide evidence that the effect of *P. copri* on weight gain in this preclinical model is diet dependent.

### Tests of *P. copri* isolates resembling MAGs Bg0018 and Bg0019

**Fig. 3:**
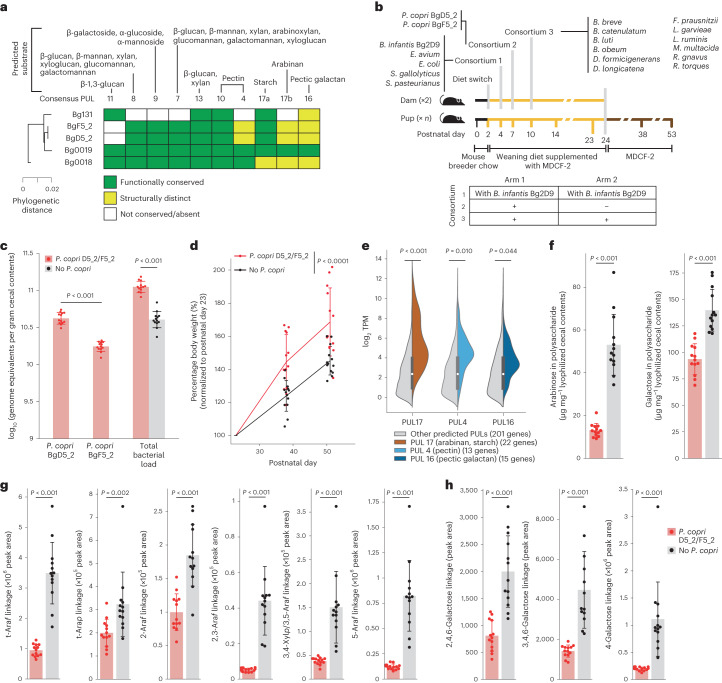
Testing the effects of pre-weaning colonization with two *P. copri* strains closely related to MAGs Bg0018 and Bg0019 on host weight gain and MDCF-2 glycan degradation. **a**, Comparison of PULs highly conserved in the two *P. copri* MAGs with their representation in the three cultured *P. copri* strains. **b**, Study design (*n* = 2 dams and 13 offspring per treatment arm). **c**, Absolute abundance of *P. copri* strains and total bacterial load in caecal contents collected at the end of the experiment (P53). Exact *P* values for comparisons of BgD5_2 and BgF5_2 and total bacterial load are 2 × 10^−5^ and 2 × 10^−^^5^, respectively. **d**, Body weights of the offspring of dams, normalized to P23. The *P* value for the group difference is *P* = 4 × 10^−^^5^ (linear mixed effects model (Methods)). **e**, GSEA of expression of PULs shared by *P. copri* BgD5_2 and BgF5_2 in the caecal contents of animals. Benjamini–Hochberg adjusted *P* values were calculated using GSEA ranking genes by their mean log_2_ TPM across the *P. copri* colonized samples, with each PUL comprising a gene set against the background of all predicted PUL genes. Violin plots show the log_2_ TPM of all genes assigned to any of the 22 predicted PULs in each isolate (*n* = 201 genes) in each of the samples, split to show homologues of consensus PUL 17 (arabinan, starch; *n* = 22 genes), PUL 4 (pectin; *n* = 13 genes) and PUL 16 (pectic galactan; *n* = 15 genes) in colour compared to the remainder of all PUL genes in grey. Internal box plots show the median (circle) and quartiles (box boundaries) for all genes assigned to PULs. *P* = 1 × 10^−4^ for PUL 17. **f**, UHPLC-QqQ-MS analysis of total arabinose and galactose in glycans present in caecal contents collected at P53. The *P* value for both arabinose and galactose is 2 × 10^−5^. **g**,**h**, UHPLC-QqQ-MS of glycosidic linkages containing arabinose (**g**) and galactose (**h**) in caecal contents. The exact *P* values for t-Ara*f*, 2-Ara*f*, 2,3-Ara*f*, 3,4-Xyl*p*/3,5-Ara*f* and 5-Ara*f* (**g**) are 2 × 10^−5^, 8 × 10^−5^, 2 × 10^−5^, 2 × 10^−5^ and 2 × 10^−5^, respectively. The exact *P* values for 2,4,6-galactose, 3,4,6-galactose and 4-galactose are 3 × 10^−5^, 2 × 10^−5^ and 2 × 10^−5^, respectively. Mean values ± s.d. are shown. *P* values were calculated using a two-sided Mann–Whitney *U* test (**c**,**f**–**h**). Each dot in **b**–**h** represents an individual animal.

We previously characterized five additional faecal *P. copri* strains that we cultured from Bangladeshi children living in Mirpur^11^. Two of these strains (BgD5_2 and BgF_2) had greater genomic similarity to MAGs Bg0018 and Bg0019 than the other isolates, including *P. copri* Bg131, as quantified by phylogenetic distance, PUL content and the representation of metabolic pathways (Fig. 3a and Supplementary Table 14a–d); for example, nine of the ten functionally conserved PULs in Bg0018/Bg0019 were present in *P. copri* BgD5_2 and BgF5_2 as were 53 of 55 carbohydrate utilization pathways (Fig. 3a and Supplementary Table 14a–d). In addition, results of in vitro growth assays conducted in defined medium supplemented with different glycans represented in MDCF-2 disclosed that strain BgF5_2 showed stronger preference than Bg131 for glycans enriched in and/or unique to MDCF-2 compared to ready-to-use supplementary food (that is, arabinan, arabinoxylan, galactan and galactomannan)^11^.

To directly determine whether pre-weaning colonization with *P. copri* strains resembling MAGs Bg0018 and Bg0019 is sufficient to promote growth and produce the metabolic effects described above, we performed an experiment whose design (Fig. 3b) was similar to our previous experiments (Fig. 1b and Extended Data Fig. 8a) but with several modifications. First, because of their greater genomic similarity to WLZ-associated MAGs Bg0018 and Bg0019, we used *P. copri* BgD5_2 and BgF5_2 in place of *P. copri* Bg131. Second, to control for differences in *B. infantis* strain used in the initial experiment (Fig. 1), both arms received *B. infantis* Bg2D9 in this ‘third’ experiment (as was the case in the second experiment described in Extended Data Fig. 8). Third, because *P. stercorea* had colonized at a lower abundance than *P. copri* and did not express CAZymes related to MDCF-2 glycans, it was not included in the second gavage mixture, which now only contained *P. copri*. Fourth, given that *B. infantis* Bg2D9 promoted pre-weaning colonization of *P. copri* in the initial experiment, we omitted the fourth gavage, previously administered at the end of the weaning period, that had included *P. copri* and *P. stercorea*. As before, the control group of animals were those that did not receive *P. copri* (*n* = 2 dams and 13 pups per treatment group).

Shotgun sequencing of DNA isolated from caecal contents collected at the time of euthanasia (P53) confirmed that animals in the experimental group had been colonized with both *P. copri* isolates as well as all other members of the defined consortia (Supplementary Table 15). Even though strain BgD5_2 grew much more poorly than BgF5_2 when cultured in defined medium^11^, in animals colonized with both isolates, the BgD5_2 strain was present at higher absolute abundance than the BgF5_2 strain (Fig. 3c) (their relative abundances were 37.8 ± 4.4% and 15.5 ± 1.0%, respectively, versus 31 ± 6.6% and 24 ± 8.0% for *P. copri* Bg131 in the experiments described in Fig. 1b and Extended Data Fig. 8a). Comparing the two groups disclosed that colonization with BgD5_2 and BgF5_2 augmented community biomass without displacing other bacteria (Fig. 3c and Supplementary Table 15b).

We observed a significantly greater increase in body weight between P23 and P53 in mice colonized with *P. copri* BgD5_2 and BgF5_2 compared to those without *P. copri* (Fig. 3d and Supplementary Table 16). The difference in the mean percentage increase in postweaning weight between the experimental and control groups (24%) was comparable to that documented in the two previous experiments shown in Fig. 1b and Extended Data Fig. 8a (25% and 13%, respectively). As in these previous experiments, the weight difference was not attributable to differences in caecal size.

**Extended Data Fig. 10:**
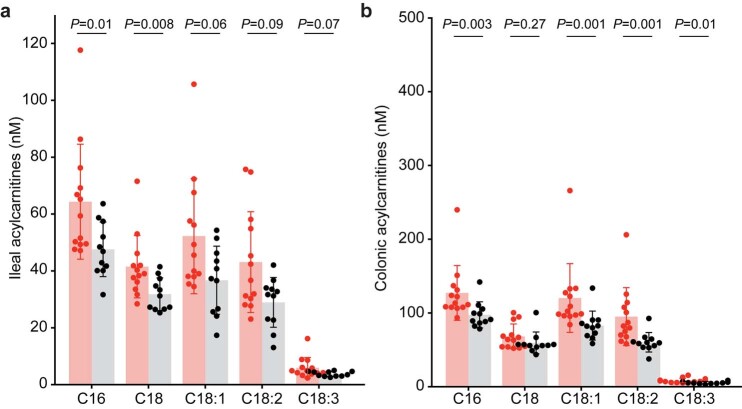
LC-MS of ileal and colonic acylcarnitines in gnotobiotic mice colonized with *P. copri* BgD5_2 and BgF5_2. (a) LC-MS of ileal acylcarnitines corresponding to soybean oil lipids. (b) LC-MS of colonic acylcarnitines corresponding to soybean oil lipids. Each dot represents an individual animal (n = 13 animals in each group). Mean values ± SD are shown. *P-*values were calculated using a two-sided Mann-Whitney U test for panel a and b.

Mass spectrometry confirmed that preweaning colonization with *P. copri* affected intestinal lipid metabolism and was a major determinant of MDCF-2 glycan degradation. Targeted LC-MS of ileal and colonic tissue revealed a significant elevation of long-chain acylcarnitines corresponding to soybean oil lipids (Extended Data Fig. 10 and Supplementary Table 17), consistent with changes observed in the experiment described in Extended Data Fig. 8. Microbial RNA-seq of caecal contents revealed that among all PUL genes, those present in the three conserved PULs with predicted arabinan/starch, pectin and pectic galactan substrates were significantly enriched for higher levels of expression (Methods; Fig. 3e and Supplementary Table 18). UHPLC-QqQ-MS of monosaccharides in glycans present in caecal contents indicated that the presence of *P. copri* BgD5_2 and BgF5_2 resulted in significantly lower levels of arabinose, consistent with our previous observations using *P. copri* Bg131, as well as galactose (a finding specific to this experiment) (Fig. 3f and Supplementary Table 19). Colonization with *P. copri* BgD5_2 and BgF5_2 also significantly lowered levels of all arabinose-containing glycosidic linkages measured, as well as three galactose-containing linkages (Fig. 3g,h and Supplementary Table 19). Together, these data indicate that the PUL content of these two isolates is associated with enhanced degradation of MDCF-2 glycans compared to the *P. copri* Bg131-containing microbial community. Targeted UPHLC-QqQ-MS measurements of all 20 amino acids and 7 B vitamins also revealed that compared to the control group, colonization with *P. copri* BgD5_2 and BgF5_2 was associated with significantly higher caecal levels of two essential amino acids (tryptophan, lysine), seven non-essential amino acids (glutamate, glutamine, aspartate, asparagine, arginine, proline, glycine) and pantothenic acid (vitamin B5) (Supplementary Table 20).

Based on all of these experiments, we concluded that (1) pre-weaning colonization with *P. copri* augments weight gain in the context of the MDCF-2 diet, (2) the presence of specific strains of this species is a major determinant/effector of MDCF-2 glycan degradation and (3) incorporating these strains into the gut community changes intestinal cellular metabolism.

## Discussion

Accessing tissue from different regions of the human intestine and extra-intestinal sites represents a major challenge when trying to characterize the mechanisms by which microbiome-targeted nutritional interventions impact the microbiota and human physiology at a molecular, cellular and systems level. In this study, we illustrate a ‘reverse translation’ strategy that can be used to address this challenge. Gnotobiotic mice were colonized with defined consortia of age- and WLZ-associated bacterial strains cultured from faecal samples collected from children living in a Bangladeshi community where the prevalence of malnutrition is high. *P. copri* was represented by cultured isolates whose genomic features, including PULs and metabolic pathways involved in carbohydrate utilization, are highly similar to MAGs associated with improved weight gain in the clinical trial. Dam-to-pup transmission of these communities occurred in the context of a sequence of diets that re-enacted those consumed by children enrolled in a clinical study of a MDCF-2. Microbial RNA-seq and targeted mass spectrometry of glycosidic linkages present in intestinal contents provided evidence that *P. copri* plays a key role in the metabolism of polysaccharides contained in MDCF-2. Consistent with this, *P. copri-*associated weight gain in the preclinical model was dependent on consumption of MDCF-2; this phenotype was not observed when a diet commonly consumed by Bangladeshi children was administered, despite comparable levels of *P. copri* colonization in the two diet contexts. snRNA-seq and targeted mass spectrometry of the intestine indicated that colonization with the consortium that contains the combination of *B. infantis* Bg2D9 and *P. copri* increases the uptake and metabolism of lipids (including those fatty acids that are most prominently represented in the soybean oil that comprises the principal lipid component of MDCF-2). Additional effects on uptake and metabolism of amino acids (including essential amino acids) and monosaccharides were predicted and in select cases validated by mass spectrometric assays. These effects on nutrient processing and energy metabolism involve proliferating epithelial progenitors in the crypts as well as their descendant lineages distributed along the villus. snRNA-seq revealed discrete spatial features of these effects, with populations of enterocytes positioned at the base-, mid- and tip regions of villi manifesting distinct patterns of differential expression of a number of metabolic functions.

Inspired by the results of the clinical trial, the goal of our ‘reverse translation’ experiments was to ascertain the impact of the presence or absence of *P. copri* in a model that emulated postnatal gut microbial community assembly and exposure to MDCF-2. The current study raises several questions that have both mechanistic and therapeutic implications. We were not able to successfully mono-colonize mice with our cultured Bangladeshi *P. copri* strains. Therefore, we could not directly test the effects of these strains in vivo on MDCF-2 glycan metabolism, weight gain and/or gut epithelial biology in the absence of other potential microbial interactions. Moreover, findings from the current study together with our findings from direct analysis of the faecal microbiomes of participants in the clinical trial^11^ indicate that degradation of MDCF-2 glycans is necessary for promoting weight gain, albeit involving the actions of downstream metabolite(s)/signalling events that remain to be fully characterized. Additional work, involving systematic manipulation of the composition of the bacterial consortia introduced into dams (and subsequently transmitted to their offspring) will be required to ascertain the extent to which *P. copri* has (1) direct effects on intestinal epithelial gene expression, host metabolism and weight gain versus (2) effects of other community members that are dependent upon its presence or absence. If *P. copri* has direct effects on the host, it remains to be determined whether the mediators of these effects are the direct products of MDCF-2 glycan metabolism or the products of other metabolic pathways in *P. copri* whose activities are regulated by biotransformation of these glycans, or other MDCF-2 components. Future studies are also needed to disambiguate the extent to which the observed effects of *P. copri* on gut epithelial carbohydrate, lipid and amino acid metabolism contribute to weight gain. The spatial features of metabolic pathway expression documented by snRNA-seq must be characterized further. This effort will be technically challenging; for example, it could require (1) documenting the distribution of *P. copri* and other community members along the crypt–villus axis, (2) advancing methods for spatial transcriptomics^29,30^ so that they can be (simultaneously) applied to both epithelial cell lineages and microbial community members and (3) using in situ mass spectrometry to directly characterize the metabolic profiles of discrete gut cell populations.

The relationship between prominent initial colonization by *B. infantis* Bg2D9 and the capacity of *P. copri* to subsequently colonize also needs further investigation. *B. infantis* Bg2D9 contains several genomic loci not represented in most other cultured *B. infantis* strains, which could enhance its ability to utilize a variety of dietary carbohydrates^13^. In principle, these loci could support increased fitness of *B. infantis* Bg2D9 in malnourished children whose consumption of breast milk is low. Given that Bangladeshi infants and young children with SAM have markedly lower levels or even completely lack *B. infantis* compared to their healthy counterparts^13^, the *B. infantis*–*P. copri* interaction documented in this preclinical study provides a rationale for testing the effects of first administering *B. infantis* Bg2D9 to individuals with SAM and subsequently MDCF-2 to restore age-appropriate microbiome configurations and promote healthy growth.

In summary, this and our companion study^11^ illustrate one approach for identifying members of a gut microbial community that function as principal metabolizers of dietary components as well as key effectors of host biological responses. The results can provide a starting point for developing microbiome-based diagnostics for stratification of populations of undernourished children who are candidates for treatment with MDCF and for monitoring their treatment responses including in adaptive clinical trial designs. Another potential return on investment for this approach is a knowledge base needed for (1) creating ‘next generation’ MDCFs composed of (already identified) bioactive glycans but from alternative food sources that may be more readily available, affordable and culturally acceptable for populations living in different geographic locales; (2) making more informed decisions about dosing of an MDCF for undernourished children as a function of their stage of development (age) and disease severity and (3) evolving policies about complementary feeding practices based on insights about how food components impact the fitness and expressed beneficial functions of growth-promoting elements of a child’s microbiome.

## Methods

### Ethics statement

The studies reported complied with all applicable ethical regulations. Bacterial strains were cultured from faecal samples collected with informed consent, under protocols approved by the International Centre for Diarrhoeal Disease Research, Bangladesh (icddr,b) Ethical Review Committee. Material transfer agreements between icddr,b and Washington University in St. Louis were established for the use of these samples. Gnotobiotic mouse experiments were performed following Institutional Animal Care and Use Committee and Institutional Biological and Chemical Safety Committee protocols approved by the Washington University Animal Studies and Environmental Health and Safety Committee.

### Bacterial genome sequencing and annotation

Monocultures of each isolate were grown overnight at 37 °C in Wilkins-Chalgren Anaerobe Broth (Oxoid, catalogue number CM0643) in a Coy Chamber under anaerobic conditions (atmosphere; 75% N_2_, 20% CO_2_ and 5% H_2_) without shaking. Cells were recovered by centrifugation (5,000 × *g* for 10 min at 4 °C). High-molecular-weight genomic DNA was purified (MagAttract HMW DNA kit, Qiagen) following the manufacturer’s protocol, and the amount was quantified (Qubit fluorometer). The sample was passed up and down through a 29-gauge needle 6–8 times, and the fragment size distribution was determined (~30 kbp; TapeStation, Agilent).

Fragmented genomic DNA (400–1,000 ng) was prepared for long-read sequencing using a SMRTbell Express Template Prep Kit 2.0 (Pacific Biosciences) adapted to a deep 96-well plate (Fisher Scientific) format. All DNA handling and transfer steps were performed with wide-bore, genomic DNA pipette tips (ART). Barcoded adapters were ligated to A-tailed fragments (overnight incubation at 20 °C), and damaged or partial SMRTbell templates were subsequently removed (SMRTbell Enzyme Cleanup Kit). High-molecular-weight templates were purified (volume of added undiluted AMPure beads = 0.45 times the volume of the DNA solution). Libraries prepared from different strains were pooled (3–6 libraries per pool). A second round of size selection was then performed; AMPure beads were diluted to a final concentration of 40% (*v*/*v*) with SMRTbell elution buffer with the resulting mixture added at 2.2 times the volume of the pooled libraries. DNA was eluted from the AMPure beads with 12 μl of SMRTbell elution buffer. Pooled libraries were quantified (Qubit), their size distribution was assessed (TapeStation) and sequenced (Sequel System, Sequel Binding Kit 3.0 and Sequencing Primer v4 (Pacific Biosystems)). The resulting reads were demultiplexed, and Q20 circular consensus sequencing reads were generated (Cromwell workflow configured in SMRT Link software). Genomes were assembled using Flye^31^ (v2.8.1) with HiFi-error set to 0.003, min-overlap set at 2,000 and other options set to default. Genome quality was evaluated using CheckM^32^ (v1.1.3) (Supplementary Table 1a, 5 and 14a).

We applied Prokka^33^ (v1.14) to identify potential open reading frames (ORFs) in each assembled genome. Additional functional annotation of these ORFs using a ‘subsystems’ approach adapted from the SEED genome annotation platform^34^ was performed as described in our companion study^11^. We assigned functions to 9,820 ORFs in 20 isolate genomes using a collection of mcSEED metabolic subsystems that capture the core metabolism of 98 nutrients/metabolites in four major categories (amino acids, vitamins, carbohydrates and fermentation products) projected over 2,856 annotated human gut bacterial genomes^35–37^. In silico reconstructions of selected mcSEED metabolic pathways were based on functional gene annotation and prediction using homology-based methods and genome context analysis. Reconstructions were represented as a binary phenotype matrix (BPM) where assignment of a ‘1’ for amino acids and B vitamins denotes a predicted prototroph and a ‘0’ denotes an auxotroph. For carbohydrates, ‘1’ and ‘0’ refer to a strain’s predicted ability or inability, respectively, to utilize the indicated mono-, di- or oligosaccharide, while for fermentation end products, a ‘1’ and ‘0’ indicate a strain’s predicted ability or inability to produce the indicated compound, respectively (Supplementary Tables 1c and 14d).

To calculate phylogenetic relationships between five *P. copri* isolates and MAGs Bg0018 and Bg0019, we first used CheckM^32^ (v1.1.3) to extract and align the amino acid sequences of 43 single-copy marker genes in each isolate or each of the two MAGs, plus an isolate genome sequence of *Bacteroides thetaiotaomicron* VPI-5482 (PATRIC accession number 226186.12). Concatenated marker gene sequences were analysed using fasttree^38^ (v2.1.10) to construct a phylogenetic tree using the Jones–Taylor–Thornton model and CAT evolution rate approximation, followed by tree rescaling using the Gamma20 optimization. The tree was subsequently processed in R using ape^39^ (v5.6-2) to root the tree with the *B. thetaiotaomicron* genome and extract phylogenetic distances between genomes, followed by ggtree^40^ (v3.2.1) for tree plotting.

The similarity between the genomes of these strains and MAGs was quantified by calculating the ANI score with pyani^41^ (ANIm (ANI calculated with the MUMmer algorithm) implementation of ANI, v0.2.10). We first calculated ANIm scores for all possible combinations between MAGs and the genomes of cultured bacterial strains and subsequently removed any MAG–strain genome combination with <10% alignment coverage^17^. For the remaining MAGs, a ‘highly similar’ genome in the collection of cultured bacterial strains was defined as having >94% ANIm score (Supplementary Table 1b). We then determined the degree of binary phenotype concordance between each genome in the collection of cultured bacterial strains and its ‘highly similar’ MAG. A binary phenotype concordance score was calculated by dividing the number of binary phenotypes^11^ shared between a cultured strain’s genome and a MAG by the total number of binary phenotypes annotated in the strain and MAG. A ‘representative MAG’ for each genome was defined as having a binary phenotype concordance score >90% (Supplementary Table 1c).

PULs were predicted based on the method described in ref. ^42^ and displayed with the Polysaccharide Utilization Loci Database (PULDB) interface^43^. PULs were placed into three categories: (1) ‘functionally conserved’ (PULs containing shared ORFs encoding the same CAZymes and SusC/SusD proteins in the same organization in their respective genomes with ≥90% amino acid identity between proteins); (2) ‘structurally distinct’ (PULs present in respective genomes but where one or more CAZymes or one or both SusC/SusD proteins are missing or fragmented in a way likely to impact function, or where extra PUL elements are present) and (3) ‘not conserved’ (PULs present in respective genomes but with mutations likely to completely compromise function, or no PUL identified).

### Colonization and husbandry

Germ-free C57BL/6J mice were maintained in plastic flexible film isolators (Class Biologically Clean) at 23 °C under a strict 12 h light cycle (lights on at 0600 h). Autoclaved paper ‘shepherd shacks’ were kept in each cage to facilitate natural nesting behaviours and provide environmental enrichment.

A weaning diet containing MDCF-2 was formulated as described in the main text. Ingredients represented in the different diet modules were combined, and the mixture was dried, pelleted and sterilized by gamma irradiation (30–50 kGy). Sterility was confirmed by culturing the pellets in brain-heart infusion medium supplemented with 0.5% yeast extract (LYBHI medium^44^) and in Wilkins–Chalgren Anaerobe Broth under aerobic and anaerobic conditions for 7 days at 37 °C followed by plating on LYBHI- and blood-agar plates. Nutritional analysis of each irradiated diet was performed by Nestlé Purina Analytical Laboratories (Supplementary Table 2c).

Pregnant C57BL/6J mice originating from trio matings were given ad libitum access to an autoclaved breeder chow (Purina Mills; Lab Diet 5021) throughout their pregnancy and until postpartum day 2. Key points about the experimental design of the gnotobiotic mouse experiments described in Figs. 1b and 3b and Extended Data Figs. 8a and 9a are as follows: (1) all bacterial strains were cultured in Wilkins–Chalgren Anaerobe Broth (except for *F. prausnitzii* which was cultured in LYBHI medium) and were collected after overnight growth at 37 °C (Supplementary Table 1a), (2) all gavage mixtures contained equivalent amounts (by OD_600_) of their constituent bacterial strains except for *F. prausnitzii* which was concentrated 100-fold before preparing the gavage mixture, (3) each bacterial consortium was administered to the postpartum dams in a volume of 200 μl using an oral gavage needle (Cadence Science; catalogue number 7901), (4) the number of dams and pups per treatment group (2 dams and 7–8 pups per treatment group for the experiment described in Fig. 1b; 4 dams and 18–19 pups per treatment group for the experiment outlined in Extended Data Fig. 8a; 2 dams and 13 pups per treatment group for the experiment illustrated in Fig. 3b; 2 dams and 12 pups per treatment group for the experiment shown in Extended Data Fig. 9a), (5) half of the bedding was replaced with fresh bedding in each cage each day from postpartum day 1 to day 14, after which time bedding was changed every 7 days, (6) diets were provided to mothers as well as to their weaning and post-weaning pups ad libitum and (7) biospecimens collected from mice when they were euthanized (without previous fasting) were snap frozen in liquid nitrogen and stored at −80 °C before use.

Pups were weighed on P23, P35 and P53 and normalized to the weight on P23. A linear mixed-effects model was used to evaluate the effect of different microbial communities on normalized mouse weight gain:

$${\rm{Normalized}}\; {\rm{weight}} \sim {\beta }_{1}\left({\rm{arm}}\right)+{\beta }_{2}\left({\rm{postnatal}}\; {\rm{day}}\right)+\left(1|{\rm{mouse}}\right)$$

### Defining the absolute abundances of bacterial strains in ileal, caecal and faecal communities

The absolute abundances of bacterial strains were determined using previously described methods with minor modifications^45,46^. In brief, 3.3 × 10^6^ cells of *Alicyclobacillus acidiphilus* DSM 14558 and 1.49 × 10^7^ cells of *Agrobacterium radiobacter* DSM 30147 (ref. ^45^) were added to each weighed frozen sample before DNA isolation and preparation of barcoded libraries for shotgun sequencing. Sequencing was performed using an Illumina NextSeq instrument. Bacterial abundances were determined by assigning reads to each bacterial genome, followed by a normalization for genome uniqueness in the context of a given community^46^. The resulting count table was imported into R^47^ (v4.0.4). We calculated the absolute abundance of a given strain *i* in sample *j* in reference to the spike-in *A. acidiphilus* (*Aa*) and *A. radiobacter* (*Ar*) genomes using the following equation:

$$\begin{array}{l}{\rm{strain}}_{i,\,j}=\left(\frac{{\rm{counts}}_{i,\,j}\times {{Aa}}\; {\rm{cells}}\; {\rm{added}}_{j}}{{{Aa}}\; {\rm{counts}}_{j}\times {\rm{sample}}\; {\rm{weight}}_{j}}+\,\frac{{\rm{counts}}_{i,j}\times {{Ar}}\; {\rm{cells}}\; {\rm{added}}_{j}}{{{Ar}}\; {\rm{counts}}_{j}\times {\rm{sample}}\; {\rm{weight}}_{j}}\right)\times 0.5\end{array}$$

The statistical significance of observed differences in the abundance of a given strain between experimental groups in ileal, caecal and faecal samples was determined by using the Kruskall–Wallis test followed by Dunn’s test for each pairwise comparison among the three arms in the experiment described in Fig. 1 or the Mann–Whitney *U* test between the two arms in experiments described in Fig. 3 and Extended Data Figs. 1b and 9. The statistical significance of differences in the composition of communities in the three arms described in Fig. 1g was determined using permutational multivariate analysis of variance (PERMANOVA)^48^ on sample projections onto principal components calculated from the log_10_ absolute abundance profiles of the 16 organisms that were not *B. infantis* or *Prevotella* species. The statistical significance of observed differences in the abundance of a given strain across different treatment groups and time was tested using a linear mixed effects model within the R packages lme4^49^ (v1.1-27) and lmerTest^50^ (v3.1-3). The change in *P. copri* absolute abundance in faecal samples during the course of the experiment was determined by a linear mixed-effects model:

$${{P.\; copri}}\; {{\rm{absolute}}\; {\rm{abundance}}} \sim {\beta }_{1}\left({\rm{arm}}\right)+{\beta }_{2}\left(\rm{postnatal}\; \rm{day}\right)+\left(1|{\rm{mouse}}\right)$$

For the experiment described in Fig. 1, 96 faecal samples were sequenced (2.2 × 10^6^ ± 1.2 × 10^5^ unidirectional 75 nt reads per sample (mean ± s.d.)), along with 20 caecal samples (1.5 × 10^6^ ± 4.2 × 10^5^ unidirectional 75 nt reads per sample) and 20 ileal samples (1.5 × 10^6^ ± 9.1 × 10^4^ unidirectional 75 nt reads per sample) (Supplementary Table 4a). For the experiment described in Extended Data Fig. 1b, 37 faecal samples were sequenced (5.8 × 10^6^ ± 1.6 × 10^6^ unidirectional 75 nt reads per sample) (Supplementary Table 6a)), while for the experiment described in Extended Data Fig. 8a, 37 caecal samples were sequenced (1.3 × 10^6^ ± 1.3 × 10^5^ unidirectional 75 nt reads per sample (Supplementary Table 11a)). For the experiment described in Fig. 3b, 26 caecal samples were sequenced (2.9 × 10^6^ ± 5.6 × 10^6^ 150 nt paired-end reads per sample (Supplementary Table 15a)).

### Microbial RNA-seq

RNA was isolated^6^ from caecal contents collected at the end of the experiment. Complementary DNA libraries were generated from isolated RNA samples using the ‘Total RNA Prep with Ribo-Zero Plus’ kit (Illumina). Barcoded libraries were sequenced (Illumina NovaSeq instrument). For the experiment described in Fig. 1b, cDNA recovered from 20 different samples of caecal contents were each sequenced to a depth of 7.8 × 10^7^ ± 9.6 × 10^6^ bidirectional 150 nt reads (mean ± s.d.) (Supplementary Table 8a). For the experiment shown in Fig. 3b, DNA from 26 different samples of caecal contents were each sequenced to a depth of 6.5 × 10^7^ ± 2.1 × 10^7^ bidirectional 150 nt reads (Supplementary Table 18a). Raw reads were trimmed by using TrimGalore^51^ (v0.6.4). Trimmed reads longer than 100 bp were mapped to reference genomes with kallisto^52^ (v0.43.0).

To analyse expression of genes from the *P. copri* and *P. stercorea* PULs described in Fig. 3e and Extended Data Fig. 4a,b, transcripts per million (TPM) values were obtained by mapping reads, using kallisto, to their genomes (Figs. 1b and 3b). TPM normalized expression was used to control for differences in library depth and gene length. For Fig. 3e and Extended Data Fig. 4a,b, log_2_ TPM with a pseudocount of 1 were visualized for all predicted PUL genes using the seaborn^53^ (v0.12.1) Python package, splitting the set to show individual PULs in colour on the right side of each violin plot against the remainder of the PUL genes shown in grey on the left side of each violin plot. Benjamini–Hochberg adjusted GSEA *P* values were calculated with fgsea^54^ (v1.20.0), ranking genes by their mean log_2_ TPM across the *Prevotella*-colonized samples in a given arm. Each PUL comprised a gene set against the background of all PUL genes from a given isolate, with minimum and maximum gene set sizes of 5 and 50 genes, respectively (Supplementary Table 8b and Supplementary Table 18b).

For the analysis described in Fig. 3e, modifications were made to account for the high fraction of genes with 100% nucleotide identity between *P. copri* BgD5_2 and BgF5_2. The kallisto index was generated to represent the set of unique genes between the two isolates by including the entire *P. copri* BgF5_2 gene set and only the subset of *P. copri* BgD5_2 genes that did not share identical sequences in *P. copri* BgF5_2 (61 out of 3,066 genes). TPMs were filtered down to the set of unique genes in the *P. copri* BgD5_2 and BgF5_2 PULs (*n* = 201 unique genes, consisting of 195 genes with identical nucleotide sequences between the two isolates and 3 unique genes from each isolate (Supplementary Table 14c)).

For abundance-normalized differential expression analysis of microbial transcripts described in Extended Data Fig. 3d, paired metagenomic and meta-transcriptomic kallisto pseudocounts were generated by mapping reads from the caecal DNA and cDNA libraries described above to the specific set of bacteria administered in the arm from which the sample was derived. To prevent skewing of library size normalization, the resulting counts matrix then underwent filtering of counts for rRNA loci predicted by Prokka^33^. MTXmodel^55^ is a generalized linear model-based approach for testing for differential expression of genes in microbial communities that controls for false positives due to differences in underlying metagenomic abundances. MTXmodel was run using the paired metagenomic and meta-transcriptomic counts tables for each organism to achieve ‘within-taxon-sum-scaling’^56^. Each pairwise comparison of arms was run using the respective sets of samples with ‘arm’ as the single fixed effect in the generalized linear model design. Transcripts with statistically significant differences in their expression (that is, ‘arm’ coefficients that were significantly different from 0) after normalizing for absolute abundance were identified (*q* value (Benjamini–Hochberg adjusted *P* value) < 0.1) after multiple hypothesis correction was applied to the entire set of transcripts from a given organism. GSEA was performed for each organism with fgsea^54^, ranking all genes surviving zero-filtering from MTXmodel differential expression testing by their estimated log_2_ fold difference. Each mcSEED metabolic pathway in each organism was used as a gene set against the background of all genes tested for differential expression, with minimum and maximum gene set sizes of 5 and 50 genes, respectively.

### Histomorphometric analysis of villus height and crypt depth

Jejunal and ileal segments were fixed in formalin and embedded vertically in paraffin; 5 μm-thick sections were prepared, and the sections were stained with haematoxylin and eosin. Slides were scanned (NanoZoomer instrument, Hamamatsu). For each animal, ten well-oriented crypt–villus units were selected from each intestinal segment for measurement of villus height and crypt depth using QuPath^57^ (v0.3.2). Measurements were performed with the investigator blinded with respect to colonization group. A two-tailed Mann–Whitney *U* test was applied to the resulting datasets.

### snRNA-seq

Jejunal segments (1.5 cm in length) were collected from mice and snap frozen in liquid nitrogen (*n* = 4 animals per treatment group (2 male mice and 2 female mice); 2 treatment groups in total). The method for extracting nuclei was adapted from a previously described protocol for the pancreas^58^. Briefly, tissues were thawed and minced in lysis buffer (25 mM citric acid, 0.25 M sucrose, 0.1% NP-40, and 1× protease inhibitor (Roche)). Nuclei were released from cells using a pestle douncer (Wheaton), washed 3 times with buffer (25 mM citric acid, 0.25 M sucrose and 1× protease inhibitor) and filtered successively through 100 μm, 70 μm, 40 μm, 20 μm and finally 5 μm diameter strainers (pluriSelect) to obtain single nuclei in resuspension buffer (25 mM KCl, 3 mM MgCl_2_, 50 mM Tris, 1 mM DTT, 0.4U μl^−1^ rNase inhibitor (Sigma) and 0.4U μl^−1^ Superase inhibitor (ThermoFisher)). Approximately 10,000 nuclei per sample were subjected to gel bead-in-emulsion generation, reverse transcription and construction of libraries for sequencing according to the protocol provided in the 3′ gene expression v3.1 kit manual (10× Genomics). Libraries were balanced, pooled and sequenced (Illumina NovaSeq S4; 3.23 × 10^8^ ± 1.39 × 10^7^ paired-end 150 nt reads per nucleus (mean ± s.d.) from jejunal samples). Read alignment, feature-barcode matrices and quality controls were processed by using the 10× Genomics CellRanger^59^ 5.0 pipeline with the flag ‘-include-introns’ to ensure that reads would be allowed to map to intronic regions of the mouse reference genome (GRCm38/mm10). Nuclei with over 2.5% reads from mitochondria-encoded genes or ribosomal protein genes were filtered out.

#### Analysis of snRNA-seq datasets

Sample integration, count normalization, cell clustering and marker gene identification was performed using Seurat 4.0^60^. Briefly, filtered feature-barcode matrices outputted from CellRanger were imported as a Seurat object using CreateSeuratObject (min.cells = 5, min.features = 200). Each sample was normalized using SCTransform^61,62^ and integrated using SelectIntegrationFeatures, PrepSCTIntegration, FindIntegrationAnchors and IntegrateData from the Seurat software package. The integrated dataset, incorporating nuclei from all samples, was subjected to unsupervised clustering using FindNeighbors (dimensions = 1:30) and FindClusters (resolution = 1) from the Seurat package, which executes a shared nearest-neighbour graph clustering algorithm to identify putative cell clusters. Cell type assignment was performed manually based on expression of reported markers^29,63,64^.

Cross-condition differential gene expression analysis was performed based on a ‘pseudobulk’ strategy: for each cell cluster, gene counts were aggregated to obtain sample-level counts; each pseudo-bulked sample served as an input for edgeR-based differential gene expression analysis^65,66^.

For NicheNet-based analysis^19^ (v1.1.0), all clusters in our snRNA-seq dataset were used as senders for crypt stem cells, proliferating transit amplifying/stem cells, villus base enterocytes, mid-villus enterocytes and villus tip enterocytes, plus goblet cells. We used the nichenet_seuratobj_aggregate (assay_oi = ‘RNA’) function with its default settings to incorporate differential gene expression information from Seurat into our NicheNet analysis and to select bona fide ligand–receptor interactions.

Compass-based in silico metabolic flux analysis^21^ (v0.9.10.2) was performed using transcripts from each of six epithelial cell clusters (crypt stem cells, proliferating transit amplifying cells, villus-base, mid-villus and villus tip enterocytes, and goblet cells). The reaction scores calculated by Compass were filtered based on (1) the confidence levels of the Recon2 reactions and (2) the completeness of information for Recon2 reaction annotations. Only Recon2 reactions that are supported by biochemical evidence (defined by Recon2 as having a confidence level of 4; ref. ^22^) and that have complete enzymatic information for the reaction were advanced to the follow-on analysis (yield: 2,075 pass filter reactions in 83 Recon2 subsystems).

We subsequently calculated a ‘metabolic flux difference’ to determine whether the presence or absence of *P. copri* affected Compass*-*based predictions of metabolic activities at the Recon2 reaction level in the six cell clusters. The ‘net reaction score’ was calculated as follows:

$$c={c}_{\rm{f}}-{c}_{\rm{r}}$$

where ${c}_{\rm{f}}$ denotes the Compass score for a given reaction in the ‘forward’ direction, and, if the biochemical reaction is reversible, ${c}_{\rm{r}}$ denotes the score for the ‘reverse’ reaction.

A Wilcoxon rank-sum test was used to test the significance of the net reaction score between the two treatment groups. *P* values from the Wilcoxon rank-sum tests were adjusted for multiple comparisons with the Benjamini–Hochberg method.

Cohen’s $d$ can be used to show the effect size of ${c}_{\rm{f}}$ or ${c}_{\rm{r}}$ for each reaction between two groups^21^ (in our case mice harbouring communities with and without *P. copri*). Briefly, Cohen’s $d$ of two groups, $j$ and $k$, was calculated based on the following two equations^67^ where *n*, *s* and *a* represent the number, the variance and the mean of the observations (in our case, the net reaction scores).

$${s}_{\rm{pool}}=\sqrt{\frac{\left({n}_{j}-1\right){s}_{j}^{2}-({n}_{k}-1){s}_{k}^{2}}{{n}_{j}+{n}_{k}-2}}$$

$$d=\frac{{a}_{j}-{a}_{k}}{{s}_{{{\mathrm{pool}}}}}$$

If both ${a}_{j}$ and ${a}_{k}$ are non-negative numbers, a positive Cohen’s $d$ indicates that the mean of group $j$ is greater than that of group $k$, whereas a negative Cohen’s $d$ means the mean of group $j$ is smaller in that comparison. The magnitude of Cohen’s $d$ represents the effect size and is correlated with the difference between the means of the two groups. Because the mean of the net subsystem scores as well as the net reaction scores could be negative, we made the following adjustments to Cohen’s *d* to preserve the concordance of sign and the order of group means. The adjusted Cohen’s *d* represents the metabolic flux difference $m$ and is defined as follows:

$$\left\{\begin{array}{c}{a}_{j} > 0{\rm{;}}\;{a}_{k} < 0{\rm{;}}\left|{a}_{j}\right| < \left|{a}_{k}\right|:m=-d\\ {a}_{j} < 0{\rm{;}}\;{a}_{k} > 0{\rm{;}}\left|{a}_{j}\right| > \left|{a}_{k}\right|:m=-d\\ {a}_{j} < 0{\rm{;}}\;{a}_{k} < 0:m=\frac{{\rm{|}}{a}_{j}{\rm{|}}-{\rm{|}}{a}_{k}{\rm{|}}}{{s}_{\rm{pool}}}\\ {\rm{other}}:m=d\end{array}\right.$$

scCODA^68^ (v0.1.8) is a Bayesian probabilistic model for detecting ‘statistically credible differences’ in the proportional representation of cell clusters, identified from snRNA-seq datasets, between different treatment conditions. This method accounts for two main challenges when analysing snRNA-seq data: (1) low sample number and (2) the compositionality of the dataset (an increase in the proportional representation of a specific cell cluster will inevitably lead to decreases in the proportional representation of all other cell clusters). Therefore, applying univariate statistical tests, such as a *t*-test, without accounting for this inherent negative correlation bias will result in reported false positives.

scCODA uses a Bayesian generalized linear multivariate regression model to describe the ‘effect’ of treatment groups on the proportional representation of each cell cluster; Hamiltonian Monte Carlo sampling is used to calculate the posterior inclusion probability of including the effect of treatment in the model. The type I error (false discovery) is derived from the posterior inclusion probability for each effect. The set of ‘statistically credible effects’ is the largest set of effects that can be chosen without exceeding a user-defined false discovery threshold *α* (*α* = 0.05 by default). We applied scCODA using default parameters, including choice of prior probability in the Bayesian model and the setting for Hamiltonian Monte Carlo sampling. The enteroendocrine cell cluster was used as the reference cluster in accordance with recommendations from the creators of scCODA to choose a cell cluster that has consistent proportional representation across samples.

### Mass spectrometry

#### UHPLC-QqQ-MS of caecal glycosidic linkages and GC-MS of short-chain fatty acids

UHPLC-QqQ-MS quantification of glycosidic linkages and monosaccharides present in caecal glycans was performed using methods described in the accompanying study^11^. Levels of short-chain fatty acid levels in caecal contents were measured by GC-MS using a procedure outlined in ref. ^6^.

#### LC-MS of acylcarnitines, amino acids and biogenic amines in host tissues

Acylcarnitines were measured in jejunum, colon, liver, gastrocnemius, quadriceps and heart muscle, plus plasma according to ref. ^69^, while 20 amino acids plus 19 biogenic amines were quantified in jejunum, liver and muscle according to methods detailed in ref. ^70^. Plasma levels of non-esterified fatty acids were measured using a UniCel DxC600 clinical analyser (Beckman Coulter).

#### Targeted mass spectrometry of caecal amino acids and B vitamins

Methods for targeted LC-QqQ-MS of amino acids and B vitamins were adapted from a previous publication^71^. Caecal samples were extracted with ice-cold methanol, and a 200 μl aliquot was dried (vacuum centrifugation; LabConco CentriVap) and reconstituted with 200 μl of a solution containing 80% methanol in water. A 2 μl aliquot of extracted metabolites was then injected into an Agilent 1290 Infinity II UHPLC system coupled with an Agilent 6470 QqQ-MS operated in positive ion dynamic multiple reaction monitor mode. The native metabolites were separated on HILIC column (ACQUITY BEH Amide, 2.1 × 150 mm, 1.7 μm particle size, Waters) using a 20 min binary gradient with constant flow rate of 0.4 ml min^−1^. The mobile phases were composed of 10 mM ammonium formate buffer in water with 0.125% formic acid (phase A) and 10 mM ammonium formate in 95% acetonitrile/H_2_O (*v*/*v*) with 0.125% formic acid (phase B). The binary gradient was as follows: 0–8 min, 91–90% B; 8–14 min, 90–70% B; 15–15.1 min, 70–91% B; 15.1–20 min, 91% B. A pool of 20 amino acids and 7 B vitamins standards with known concentrations (amino acid pool: 0.1 ng ml^−1^ to 100 μg ml^−1^; B vitamin pool: 0.01 ng ml^−1^ to 10 μg ml^−1^) was injected along with the samples as an external calibration curve for absolute quantification.

### Statistics and reproducibility

Dams were randomly assigned to different treatment groups in all experiments. The sample size of treatment groups in all of the experiments was determined by the size of the litter. No statistical methods were used to pre-determine sample sizes, but our sample sizes are similar to those reported in previous publications^6,13^. Both sexes of the offspring were used. No data generated from the experiment were excluded. The statistical tests used in this study do not require data to be normally distributed and do not assume equal variance. While data collection and analysis were not performed blinded to the experimental group, the initial experiment and validation experiments were performed independently by different members of our team. The snRNA-seq analysis and the subsequent targeted metabolomics quantification were also conducted by different scientists.

### Biological materials

Human specimens and bacterial strains cultured from faecal samples collected from Bangladeshi children are the property of icddr,b. Material transfer agreements exist between icddr,b and Washington University for the use of these samples. Requests for materials should be made to J.I.G.

### Reporting summary

Further information on research design is available in the Nature Portfolio Reporting Summary linked to this article.

## Supplementary information

Reporting Summary

Supplementary Tables Supplementary Tables 1–20.

## Data Availability

Microbial community and long-read bacterial strain genome sequencing datasets, bacterial genome assemblies and microbial RNA-seq and snRNA-seq datasets have been deposited in the National Center for Biotechnology Information Sequence Read Archive (SRA) under study accession number PRJNA1067830. UHPLC-QqQ-MS datasets are available in Glycopost under study accession number GPST000392.

## Code availability

All software used were from publicly available sources.
