# Supplementary material for: Prevotella copri and microbiota members mediate the beneficial effects of a therapeutic food for malnutrition
Source: Nat Microbiol. 2024 Mar 19;9(4):922–37. doi: 10.1038/s41564-024-01628-7 (PMC10994852; doi:10.1038/s41564-024-01628-7)
Supplement: Supplementary file 1 — Reporting Summary [file 41564_2024_1628_MOESM1_ESM.pdf]

nature portfolio

Corresponding author(s): Jeffrey Gordon

Last updated by author(s): Nov 20, 2023

Reporting Summary

Nature Portfolio wishes to improve the reproducibility of the work that we publish. This form provides structure for consistency and transparency in reporting. For further information on Nature Portfolio policies, see our Editorial Policies and the Editorial Policy Checklist.

Statistics

For all statistical analyses, confirm that the following items are present in the figure legend, table legend, main text, or Methods section.

n/a	Confirmed	
☐	☒	The exact sample size (n) for each experimental group/condition, given as a discrete number and unit of measurement
☐	☒	A statement on whether measurements were taken from distinct samples or whether the same sample was measured repeatedly
☐	☒	The statistical test(s) used AND whether they are one- or two-sided<br>*Only common tests should be described solely by name; describe more complex techniques in the Methods section.*
☐	☒	A description of all covariates tested
☐	☒	A description of any assumptions or corrections, such as tests of normality and adjustment for multiple comparisons
☐	☒	A full description of the statistical parameters including central tendency (e.g. means) or other basic estimates (e.g. regression coefficient) AND variation (e.g. standard deviation) or associated estimates of uncertainty (e.g. confidence intervals)
☐	☒	For null hypothesis testing, the test statistic (e.g. F, t, r) with confidence intervals, effect sizes, degrees of freedom and P value noted<br>*Give P values as exact values whenever suitable.*
☐	☒	For Bayesian analysis, information on the choice of priors and Markov chain Monte Carlo settings
☒	☐	For hierarchical and complex designs, identification of the appropriate level for tests and full reporting of outcomes
☐	☒	Estimates of effect sizes (e.g. Cohen's d, Pearson's r), indicating how they were calculated

Our web collection on statistics for biologists contains articles on many of the points above.

Software and code

Policy information about availability of computer code

Data collection	No software was used for data collection
Data analysis	All software packages used are described in Methods and are publicly available; they include ape (v5.6-2), bcl2fastq(v1.8.4), Trim Galore(v0.6.4), Kallisto (v0.43.0), MTXmodel (v1.2.3), edgeR (v3.32.1), seaborn (v.0.12.1), fgsea (v.1.20.0), QuPath (v0.3.2), CellRanger (v5.0), Seurat (v4.0), NicheNet (v1.1.0), Compass (v0.9.10.2), scCODA (v0.1.8), Flye (v2.8.1), CheckM (v1.1.3), Prokka (v1.14), and pyani (v0.2.10). Code detailing the steps in the MAG assembly workflow and analyses of microbial RNA-Seq and glycan datasets are available from GitLab (https://gitlab.com/hibberdm/hibberd_webber_et_al_mdcf_poc_mags) and have been accessioned at Zenodo (DOI:10.5281/zenodo.8000098). Code for annotation of bacterial genes and prediction of metabolic phenotypes is available from GitHub (https://github.com/rodionovdima/PhenotypePredictor) and has been accessioned at Zenodo (DOI: 10.5281/zenodo.10049439).

For manuscripts utilizing custom algorithms or software that are central to the research but not yet described in published literature, software must be made available to editors and reviewers. We strongly encourage code deposition in a community repository (e.g. GitHub). See the Nature Portfolio guidelines for submitting code & software for further information.

Data

Policy information about availability of data

All manuscripts must include a data availability statement. This statement should provide the following information, where applicable:

- Accession codes, unique identifiers, or web links for publicly available datasets
- A description of any restrictions on data availability
- For clinical datasets or third party data, please ensure that the statement adheres to our policy

Microbial community and long-read bacterial strain genome sequencing datasets, bacterial genome assemblies, and microbial RNA-Seq and snRNA-Seq datasets

have been deposited in the NCBI Sequence Read Archive (SRA) under study accession number PRJNA1067830. UHPLC-QqQ-MS datasets are available in Glycopost under study accession number GPST000392.

Field-specific reporting

Please select the one below that is the best fit for your research. If you are not sure, read the appropriate sections before making your selection.

☒ Life sciences ☐ Behavioural & social sciences ☐ Ecological, evolutionary & environmental sciences

For a reference copy of the document with all sections, see nature.com/documents/nr-reporting-summary-flat.pdf

Life sciences study design

All studies must disclose on these points even when the disclosure is negative.

Sample size	The number of animals studied per treatment group was based on the number of pups born to each litter. Sample sizes were based off past preclinical studies on effects of MDCF prototypes on microbial communities and the effects of B. infantis on weight gain (ref. 6 and 13). The exact number of animals used in each experiment is noted in the main text, figure legends and methods.
Data exclusions	No data were excluded
Replication	Two similar validation experiments were performed (Extended Data Fig. 8 and Fig. 3) which reproduced the key findings of weight gain with P. copri colonization and changes in host and microbial community metabolism. The experiment described in Extended Data Fig. 1 was repeated independently twice confirming the colonization dependency of P. copri on B. infantis.
Randomization	Before initiation of the gnotobiotic experiments, dams were randomly assigned to each treatment group. Processing of samples from gnotobiotic mice alternated experimental groups to reduce potential technical biases.
Blinding	All data were generated from all samples without knowledge of treatment group

Reporting for specific materials, systems and methods

We require information from authors about some types of materials, experimental systems and methods used in many studies. Here, indicate whether each material, system or method listed is relevant to your study. If you are not sure if a list item applies to your research, read the appropriate section before selecting a response.

Materials & experimental systems

n/a	Involved in the study	
☒	☐	Antibodies
☒	☐	Eukaryotic cell lines
☒	☐	Palaeontology and archaeology
☐	☒	Animals and other organisms
☐	☒	Human research participants
☒	☐	Clinical data
☒	☐	Dual use research of concern

Methods

n/a	Involved in the study	
☒	☐	ChIP-seq
☒	☐	Flow cytometry
☒	☐	MRI-based neuroimaging

Animals and other organisms

Policy information about studies involving animals; ARRIVE guidelines recommended for reporting animal research

Laboratory animals	Germ-free C57BL/6J mice and their pups were used. Animals were euthanized at P42 for experiments testing colonization dependency of P. copri and B. infantis and at P53 for all other experiments.<br><br>Germ-free C57BL/6J mice were maintained in plastic flexible film isolators (Class Biologically Clean Ltd) at 23 ºC under a strict 12-hour light cycle (lights on at 0600h). Autoclaved paper 'shepherd shacks' were kept in each cage to facilitate natural nesting behaviors and provide environmental enrichment. For all experiments, pregnant C57BL/6J mice originating from trio matings were given ad libitum access to an autoclaved breeder chow (Purina Mills; Lab Diet 5021) throughout their pregnancy and until postpartum day 2. Mice then underwent the sequence of diet changes described in each experiment's design with ad libitum food access.
Wild animals	No wild animals were used
Field-collected samples	No field-collected samples were used
Ethics oversight	All gnotobiotic mouse experiments were performed following IACUC and IBC protocols that were approved by the Washington University Animal Studies and Environmental Health and Safety Committee.

Note that full information on the approval of the study protocol must also be provided in the manuscript.

Human research participants

Policy information about studies involving human research participants

Population characteristics	The human biospecimens used for the gnotobiotic mouse experiments described in this manuscript are from an already reported clinical study whose population characteristics are detailed in ref. 6 (N Engl J Med 2021 (DOI: 10.1056/ NEJMoa2023294).
Recruitment	The human biospecimens used for the gnotobiotic mouse experiments described in this manuscript are from an already reported clinical study whose protocols for participant recruitment are described in ref. 6 (N Engl J Med 2021 (DOI: 10.1056/ NEJMoa2023294).
Ethics oversight	The human biospecimens used for the gnotobiotic mouse experiments described in this manuscript are from an already reported clinical study that was approved by the ethical review committee of the International Centre for Diarrhoeal Disease Research, Bangladesh as described in ref. 6 (N Engl J Med 2021 (DOI: 10.1056/NEJMoa2023294).

Note that full information on the approval of the study protocol must also be provided in the manuscript.
